# Divergent Survival Outcomes Associated with Elevated Branched-Chain Amino Acid Levels among Older Adults with or without Hypertension and Diabetes: A Validated, Prospective, Longitudinal Follow-Up Study

**DOI:** 10.3390/biom13081252

**Published:** 2023-08-16

**Authors:** Erik Fung, Kwan Hung Ng, Timothy Kwok, Leong-Ting Lui, Saranya Palaniswamy, Queenie Chan, Lee-Ling Lim, Petri Wiklund, Suyi Xie, Cheryl Turner, Amany K. Elshorbagy, Helga Refsum, Jason C. S. Leung, Alice P. S. Kong, Juliana C. N. Chan, Marjo-Riitta Järvelin, Jean Woo

**Affiliations:** 1 Department of Medicine and Therapeutics, Faculty of Medicine, The Chinese University of Hong Kong, Hong Kong SAR, China; 2 Gerald Choa Cardiac Research Centre and Laboratory for Heart Failure + Circulation Research, Li Ka Shing Institute of Health Sciences, Prince of Wales Hospital, Hong Kong SAR, China; 3 Neural, Vascular, Metabolic Biology Programme, and Ministry of Education Key Laboratory for Regenerative Medicine, School of Biomedical Sciences, The Chinese University of Hong Kong, Hong Kong SAR, China; 4 Division of Cardiology, Department of Medicine, School of Medicine, The Chinese University of Hong Kong, Shenzhen 518172, China; 5 Department of Epidemiology and Biostatistics, School of Public Health, Imperial College London, London W2 1PG, UK; 6 CUHK Jockey Club Centre for Osteoporosis Care and Control, Faculty of Medicine, The Chinese University of Hong Kong, Hong Kong SAR, China; 7 Center for Life Course Health Research, Faculty of Medicine, University of Oulu, 90014 Oulu, Finland; 8 Department of Medicine, Faculty of Medicine, University of Malaya, Kuala Lumpur 50603, Malaysia; 9 Asia Diabetes Foundation, Shatin, Hong Kong SAR, China; 10 Faculty of Sport and Health Sciences, University of Jyväskylä, 40014 Jyväskylä, Finland; 11 The Exercise Translational Medicine Center and Shanghai Center for Systems Biomedicine, Shanghai Jiao Tong University, Shanghai 200240, China; 12 Department of Pharmacology, University of Oxford, Oxford OX1 3QT, UK; 13 Department of Physiology, Faculty of Medicine, University of Alexandria, Alexandria 21526, Egypt; 14 Department of Public Health and Primary Healthcare, School of Public Health, Imperial College London, London W2 1PG, UK; 15 Department of Nutrition, Institute of Basic Medical Sciences, University of Oslo, 0372 Oslo, Norway; 16 Hong Kong Institute of Diabetes and Obesity, The Chinese University of Hong Kong, Hong Kong SAR, China; 17 Li Ka Shing Institute of Health Sciences, Faculty of Medicine, The Chinese University of Hong Kong, Hong Kong SAR, China; 18 Department of Life Sciences, College of Health and Life Sciences, Brunel University London, Kingston Lane, Uxbridge UB8 3PH, UK; 19 Unit of Primary Health Care, Oulu University Hospital, 90014 Oulu, Finland; 20 CUHK Jockey Club Institute of Ageing, The Chinese University of Hong Kong, Hong Kong SAR, China

**Keywords:** branched-chain amino acid, hypertension, diabetes mellitus, mortality hazard, older adults

## Abstract

Branched-chain amino acids are critical metabolic intermediates that can indicate increased risk of cardiometabolic disease when levels are elevated or, alternatively, suggest sufficient mitochondrial energy metabolism and reserve in old age. The interpretation of BCAA levels can be context-dependent, and it remains unclear whether abnormal levels can inform prognosis. This prospective longitudinal study aimed to determine the interrelationship between mortality hazard and fasting serum BCAA levels among older men and women aged ≥65 years with or without hypertension and diabetes mellitus. At baseline (0Y), fasting serum BCAA concentration in 2997 community-living older men and women were measured. Approximately 14 years later (14Y), 860 study participants returned for repeat measurements. Deaths were analysed and classified into cardiovascular and non-cardiovascular causes using International Classification of Diseases codes. Survival analysis and multivariable Cox regression were performed. During a median follow-up of 17Y, 971 (78.6%) non-cardiovascular and 263 (21.4%) cardiovascular deaths occurred among 1235 (41.2%) deceased (median age, 85.8 years [IQR 81.7–89.7]). From 0Y to 14Y, BCAA levels declined in both sexes, whereas serum creatinine concentration increased (both *p* < 0.0001). In older adults without hypertension or diabetes mellitus, the relationship between mortality hazard and BCAA level was linear and above-median BCAA levels were associated with improved survival, whereas in the presence of cardiometabolic disease the relationship was U-shaped. Overall, adjusted Cox regression determined that each 10% increment in BCAA concentration was associated with a 7% (*p* = 0.0002) and 16% (*p* = 0.0057) reduction in mortality hazard estimated at 0Y and 14Y, respectively. Our findings suggested that abnormally high or low (dyshomeostatic) BCAA levels among older adults with hypertension and/or diabetes mellitus were associated with increased mortality, whereas in those with neither disease, increased BCAA levels was associated with improved survival, particularly in the oldest-old.

## 1. Introduction

The disproportionate expansion in the number of people aged 65 years and above worldwide has resulted in global population ageing [1]. Ageing is associated with a rise in noncommunicable diseases, particularly cardiovascular disease, cancer, chronic respiratory disease and diabetes mellitus, which exert pressure on healthcare services and consume resources [2,3]. Hypertension and diabetes are among the most important lifetime risk factors for morbidity and mortality [4,5,6,7,8], with over 500 million people worldwide afflicted with either or both [7,8]. A thorough understanding of their shared pathophysiology may yield opportunities to improve the risk stratification and management of cardiometabolic disorder.

Branched-chain amino acids (BCAAs), including leucine, isoleucine and valine, are essential amino acids required for mitochondrial energy metabolism, biogenesis and protein biosynthesis under homeostatic conditions [9,10,11]. BCAA levels are abnormally elevated in the blood circulation of patients with hypertension and/or diabetes [9,10,11]. In cardiometabolic diseases, BCAA conversion into acyl-CoA derivatives (e.g., acetyl-CoA, succinyl-CoA) is suppressed, leading to the accumulation of intracellular BCAAs and metabolic shunting to other pathways for energy generation [9,10,11]. Elevated circulating BCAA levels are associated with subclinical as well as clinically important cardiovascular diseases and adverse outcomes, including hypertension [12], cardiomyopathy [13], heart failure [14,15], acute myocardial infarction [15,16,17], and stroke [17]. Moreover, recent animal studies have shown that BCAA supplementation may be associated with harm and reduced lifespan [18].

Although increased BCAA levels have been associated with cardiometabolic dysfunction and disease, a Belgian population-based longitudinal study of participants aged 50–55 years reported that higher circulating levels of leucine and valine were associated with improved ventricular diastolic function [19], lending support to preclinical rodent studies that dietary BCAA supplementation benefitted cardiomyocyte survival and myocardial function, and prolonged lifespan [20,21]. Despite having important roles in energy metabolism and serving as biomarkers for cardiometabolic risks [22,23], it remained unclear whether BCAAs could provide prognostic information on non-cardiovascular mortality in human.

Epidemiological and cohort studies supported the concept that high circulating BCAA levels were associated with hypernutrition, obesity and cardiometabolic disease in childhood and middle adulthood, whereas diminished BCAA levels in older adults reflect an overall catabolic state and decline in physiological reserve [24]. In older adults in whom physiological reserve and nutrition are crucial for immunity, the maintenance of homeostasis and survival, low levels of BCAA have been associated with adverse clinical outcomes in specific subsets of older adults [24]. Several published cohort studies focusing on either sex (e.g., Women’s Health Study [17,25], Australian Men in the Concord Health and Ageing in Men Project [26]) have reported important associations between BCAA levels and cardiometabolic derangements. Few prospective longitudinal studies have analysed the long-term interrelationships between BCAAs, hypertension and diabetes in both older men and women over a decade. In a cohort study of 918 community-living Australian older men who were followed for 5 years, Le Couteur et al. reported that BCAA levels in the lowest quartile were associated with the highest risk for incident mortality and adverse major adverse cardiovascular outcomes [26]. Although the study did not include female participants, it suggested that BCAAs could be informative for prognosis. However, the long-term prognostic significance and characteristics of BCAA levels in both sexes and the concurrent impact of hypertension and diabetes remain poorly characterised in old age and towards end of life [12,13,14,15,16,17,19,22,23,24], as most studies on adverse outcomes have focused on participants with average ages in the range of 51–69 years [12,13,14,15,16,17].

The Hong Kong population has among the longest life expectancy in the world, and include survivors of wars, social turmoil, and famine [27,28,29]. The surviving older adult population of Hong Kong is an invaluable resource for gaining insight into the long-term pathophysiological alteration in BCAA metabolism over an extended period until the end of life. In this prospective, longitudinal cohort study of 2997 community-living older men and women in Hong Kong, we quantified BCAA levels at baseline (0Y) and 14 years (14Y); characterised the interrelationship of BCAAs, hypertension and diabetes; and determined, using 0Y and 14Y BCAA levels, the incident risks of cardiovascular and non-cardiovascular mortality during a median follow-up of 17 years.

## 2. Methods

### 2.1. Study Cohort

The study population included 2997 community-living men and women of the Mr & Ms Os Cohort (Hong Kong) who were aged 65 years or above at the time of recruitment during 2003–2005 (baseline, 0Y) [29]. Approximately 14 years later (14Y), the study participants were invited back for follow-up evaluations. Written informed consent was obtained from all participants at study entry. The study received ethical approval (CREC no. 2003.102) from the local institutional review board, The Chinese University of Hong Kong–New Territories East Cluster Clinical Research Ethics Committee, and complied with the Declaration of Helsinki.

### 2.2. Definitions of Hypertension, Diabetes Mellitus, Frailty, and Estimated Glomerular Filtration Rate

Information about participants’ status of hypertension and diabetes at baseline were obtained from self-reported standardised questionnaires and from diagnoses documented in medical records. Hypertension was diagnosed if systolic blood pressure was persistently ≥140 mmHg or diastolic blood pressure ≥ 90 mmHg [30]. Diabetes mellitus was diagnosed according to the World Health Organization and International Diabetes Federation criteria for the disease: fasting plasma glucose ≥ 7.0 mmol/L, 2 h plasma glucose ≥ 11.1 mmol/L, and/or HbA1c ≥ 6.5% [31]. The study participants’ use of antihypertensive and/or hypoglycaemic drugs at study entry was recorded from clinical charts and/or the electronic medical record system of the Hospital Authority of Hong Kong (Table 1). Frailty was assessed using Rockwood’s frailty index, a cumulative deficit approach of assessment that quantifies frailty by calculating the ratio of the actual number of diseases or disorders present to the total number (typically, between 30 and 70) assessed [32]. Estimated glomerular filtration rate (eGFR) was calculated using the Chronic Kidney Disease Epidemiology Collaboration (CKD-EPI) equation [33].

### 2.3. Phlebotomy, Serum Processing, and Sample Storage

Phlebotomy was performed on 2997 participants at 0Y and on 860 participants at 14Y when they returned for follow-up evaluations. Within 2–3 h of phlebotomy at both time points, 0Y and 14Y, fasting blood samples were centrifuged and serum was isolated. Serum specimens were stored at −80 °C until measurement of BCAA levels (see below for details). For the 0Y time point, samples were collected at study entry during 2003–2005, and stored at −80 °C until measurement of BCAA levels in 2016 (approximately 12 years after collection) at the Department of Pharmacology, University of Oxford, Oxford, UK. For the 14Y time point, serum specimens were collected during 2019–2021, stored at −80 °C and assayed for BCAAs in 2022 (approximately 2 years after collection) at Nightingale Health Limited, Helsinki, Finland. The samples fulfilled quality control analysis prior to processing by the respective laboratories.

### 2.4. Quantification of Serum Concentration of Branched-Chain Amino Acids and Creatinine

Fasting serum collected at 0Y were quantified for BCAAs using liquid chromatography–mass spectrometry in the Department of Pharmacology, University of Oxford [34]. Samples were mixed with isotopically labelled internal standards, disulphides were reduced using dithioerythritol, and 5-sulfosalicyclic acid was used for protein precipitation. An aqueous solution of 0.5% formic acid and 0.3% heptafluorobutyric acid were added to the extract prior to analysis using a Shimadzu LC-20ADXR Prominence LC system (Kyoto, Japan), Sciex QTRAP5500 MS with a Turbo V ion source, and a TurboIonspray probe (Framingham, MA, USA). LC separation was performed using a Phenomenex Kinetex Core Shell C18 (100 mm × 4.6 mm, 2.6 μm) LC column (Torrance, CA, USA) with an aqueous solution of 0.5% formic acid and 0.3% heptafluorobutyric acid, and an acetonitrile gradient mobile phase. Multiple reaction monitoring for positive ions was used for detection. Quantification was done by comparing the corresponding linear calibration curves of the peak area ratios of the analytes with the internal standard. Spectral data were matched against standards and reference libraries to determine the identities of compounds. For fasting serum collected at 14Y, samples were analysed for BCAAs using a Bruker AVANCE III 600 MHz nuclear magnetic resonance spectrometer in a proprietary pipeline by Nightingale Health Limited [35]. Previous studies have demonstrated highly significant correlations (*p* < 0.001) between mass spectrometry and nuclear magnetic resonance spectroscopy in the quantification of blood levels of metabolites [36] For total BCAAs levels, a previously published large-scale UK Biobank study that used Nightingale NMR profiling listed a % coefficient of variation (%CV) of approximately 5–6% [37].

### 2.5. Ascertainment of Deaths

Death was ascertained by querying the Deaths Registries of Hong Kong and reviewing medical records. The last query was completed in June 2020. International Classification of Diseases (ICD) codes were collected and reviewed to categorise causes of deaths as being cardiovascular (Table S1) or non-cardiovascular (Table S2).

### 2.6. Data and Statistical Analysis

Distribution of continuous variables were assessed for normality using the Shapiro–Wilk test. Average values for data that followed a normal distribution were shown as mean ± standard deviation (SD). Nonparametric data were shown as median with interquartile range (IQR). BCAA concentration was log-transformed (log(BCAA)) where appropriate to adjust for skewing. Student’s *t* test was used to compare groups with normally distributed data whereas the Mann–Whitney U test was used for nonparametric testing. Partial correlation adjusted for age and sex was used to compare associations between BCAAs. Kaplan–Meier analysis was used for an estimation of survival probability; both log-rank test and pairwise *t* test results were calculated. In multivariable Cox proportional hazards model, independent variables were regressed on all-cause, cardiovascular or non-cardiovascular mortality and adjusted for covariates. The follow-up period for modelling adjusted hazard ratios (HRs) was calculated from the date of study entry until occurrence of death. To convert the HR of log(BCAA) from an exponential increase (e = 2.718 times) to 1.1-times increase, the value was transformed by $HR^{\prime }=HR^{\ln(1.1)}$. To determine if a nonlinear relationship existed between variables, a linear model and a restricted cubic spline model with 3 knots were computed for each stratified group. The best-fit model was determined by the lowest Akaike information criterion (AIC). Nonlinearity was indicated by a *p*-value < 0.05 for the best-fit restricted cubic spline model. Analyses were done using R statistical programming software version 4.2.1 (R Foundation for Statistical Computing, Vienna, Austria. http://www.r-project.org (accessed on 1 June 2023)). The “rms” package was used to generate restricted cubic spline models. A two-tailed *p* < 0.05 was considered to be statistically significant.

## 3. Results

### 3.1. Baseline Characteristics of Longitudinal Study Cohort

The median age of 2997 community-living older adults (47.5% male) in this cohort was 71 (IQR 68–75) years at baseline. Among them, 1282 (42.8%) and 425 (14.2%) had hypertension and diabetes mellitus, respectively (Table 1). Hypertension was associated with older age (median 72 [IQR 68–75] vs. 71 [IQR 68–75] years, *p* < 0.0001), increased frailty (frailty index 0.13 [IQR 0.11–0.19] vs. 0.06 [IQR 0.04–0.11], *p* < 0.0001), higher BMI (24.4 [IQR 22.4–26.4] vs. 23 [IQR 21.2–25.3] kg/m^2^, *p* < 0.0001), higher serum creatinine concentration (72.8 [IQR 60.7–87.8] vs. 68.4 [IQR 57.1–81.2] µmol/L, *p* < 0.0001), the presence of CKD (defined as eGFR <60; 127 (9.9%) vs. 62 (3.6%), *p* < 0.0001), lower physical activity assessed by PASE (84.7 [IQR 62.2–110.4] vs. 86.3 [676.6–115.3], *p* < 0.01), and higher rates of diabetes mellitus (22.5% vs. 7.9%, *p* < 0.0001), stroke (6.4% vs. 2.0%, *p* < 0.0001) and heart failure (5.7% vs. 2.5%, *p* < 0.0001), compared with non-hypertension. Diabetes mellitus was associated with a higher BMI (24.3 [IQR 22.4–26.4] vs. 23.5 [IQR, 21.5–25.6], *p* < 0.0001), increased frailty (frailty index, 0.17 [IQR 0.11–0.21] vs. 0.09 [IQR 0.04–0.13], *p* < 0.0001), and high rates of cardiovascular comorbidities but not COPD or CKD (Table 1). No difference in the level of physical activity (assessed by PASE), eGFR or CKD status was observed between diabetic and non-diabetic study participants (Table 1). The use of antihypertensive and hypoglycaemic agents was significantly greater in older adults with hypertension and diabetes mellitus, respectively (Table 1). Individuals with hypertension and diabetes mellitus had higher levels of total BCAA levels than their non-hypertensive and non-diabetic counterparts (HT vs. no HT, *p* < 0.0001 and DM vs. no DM, *p* < 0.0001) (Table 2). Men had a consistently and significantly higher serum concentration of BCAAs, compared with women (Table S3). Within-individual levels of isoleucine, leucine and valine were strongly correlated (isoleucine vs. leucine, r = 0.878; isoleucine vs. valine, r = 0.838; leucine vs. valine, r = 0.867; all *p* < 0.0001 by partial correlation adjusted for age and sex), and justified their combination as a single variable [12].

### 3.2. Interval Measurements of Serum Concentration of Branched-Chain Amino Acids and Creatinine at Baseline and at 14 Years

**Figure 1:**
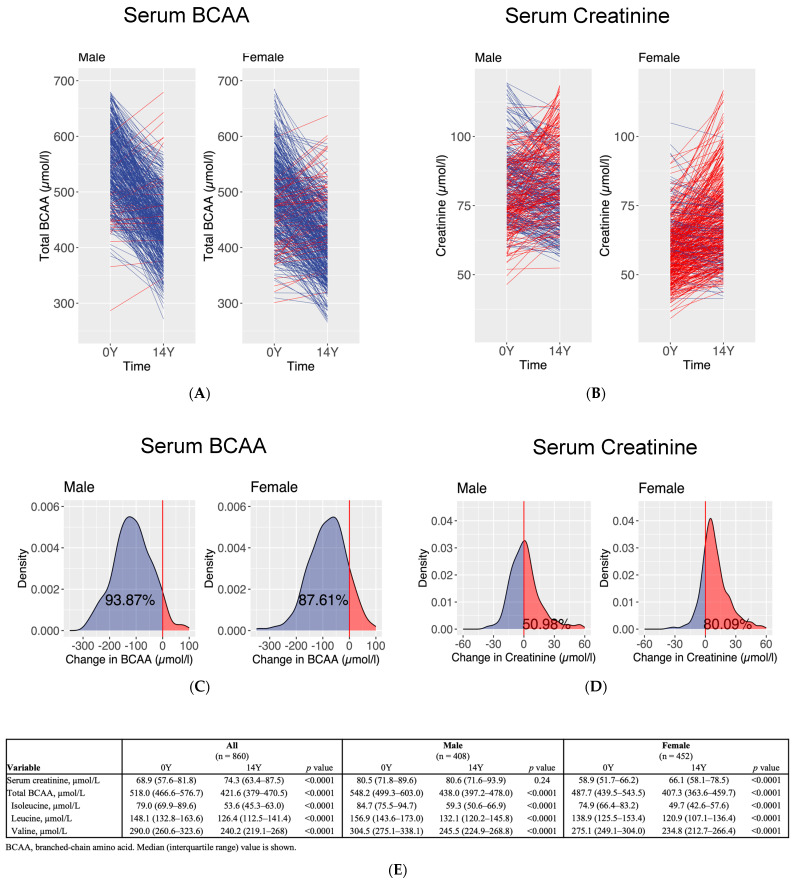
Interval measurement of fasting serum concentrations of (**A**) BCAA and (**B**) creatinine at baseline (0Y) and 14 years (14Y) in male and female study participants. Density plots show changes in (**C**) BCAA and (**D**) creatinine concentrations from 0Y to 14Y. Summary table shows the (**E**) mean concentrations of BCAA and creatinine at 0Y and 14Y in male and female participants. Decreases and increases are indicated by blue and red colours, respectively.

At 14Y, 860 participants (median age 83 years [IQR 81–86], 47.4% male) were available for a repeat measurement of fasting serum BCAA levels. From 0Y to 14Y, BCAA levels decreased irrespective of sex or baseline status of hypertension and diabetes (Table 2 and Figure 1). Total BCAA levels decreased from 514.3 (IQR 463.2–574.6) to 421.6 (IQR 379.0–470.5) µmol/L, whereas serum creatinine concentration increased from 70.3 (IQR 58.2–84.3) to 74.3 (IQR 63.4–87.5) µmol/L. Hypertension and diabetes were significantly associated with higher serum creatinine concentration compared with their counterparts without the respective conditions (78.2 [IQR 65.8–93.9] vs. 71.5 [IQR 62.0–83.6] µmol/L, *p* < 0.0001 and 81.0 [IQR 68.0–98.2] vs. 73.0 [IQR 62.8–85.7] µmol/L, *p* < 0.0001, respectively) (Table 2). A greater proportion of women (80.1%) than men (51.0%) had a rise in serum creatinine concentration between 0Y and 14Y (Figure 1B).

### 3.3. Survival Analysis during a Median Follow-Up of 17 Years

**Figure 2:**
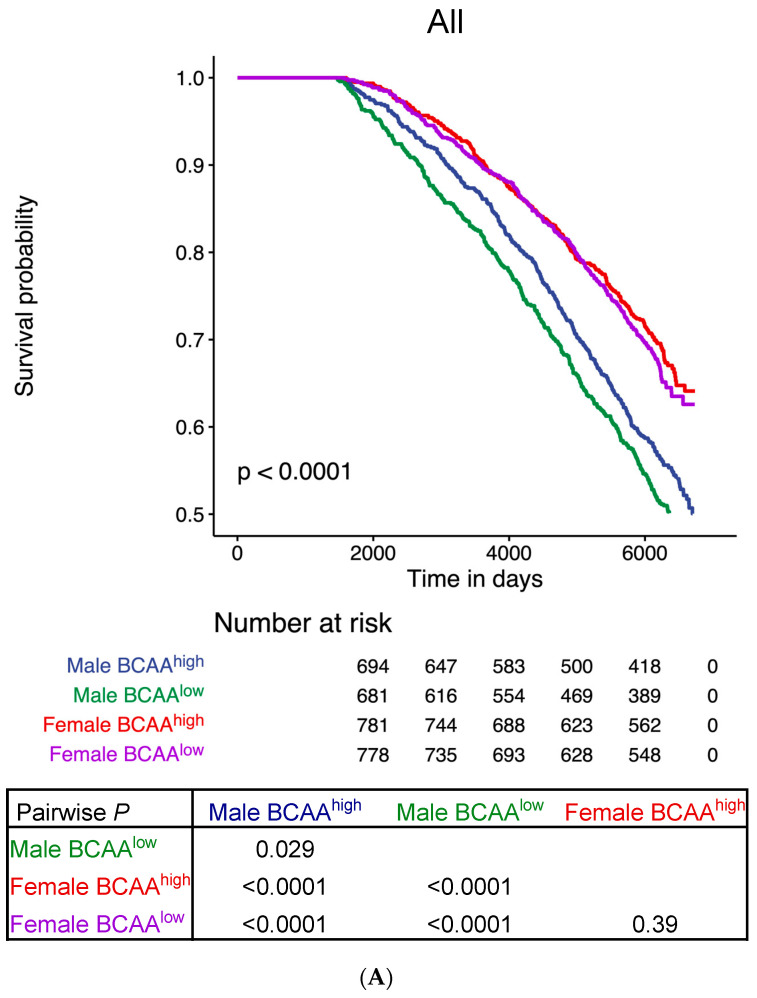

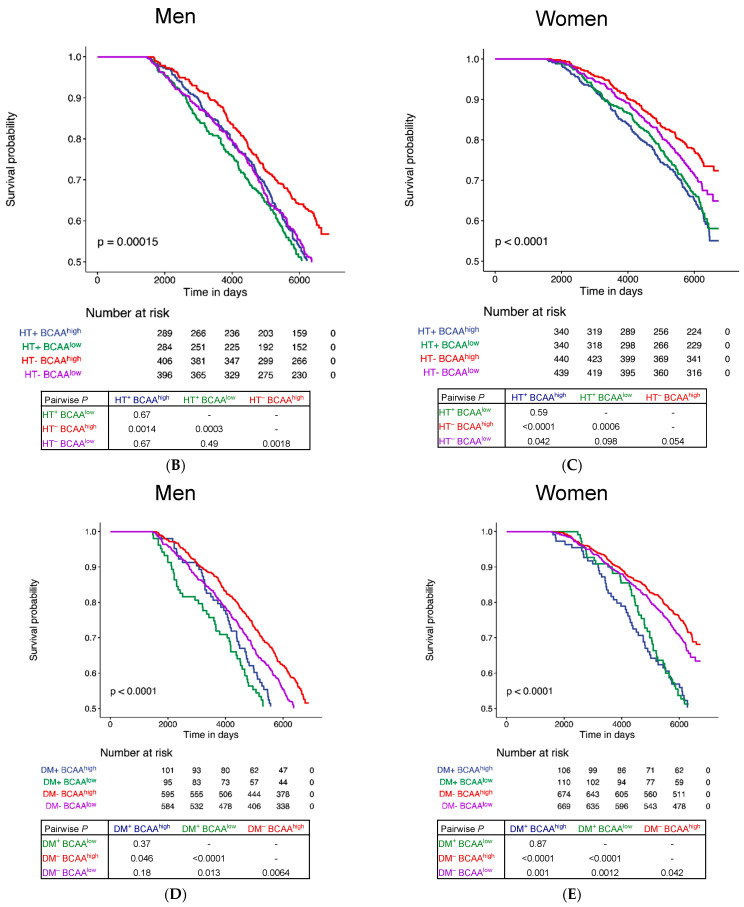
Kaplan–Meier survival analysis of the cohort as stratified by BCAA levels and cardiometabolic status. All-cause mortality was stratified by (**A**) above (BCAA^+^) and below (BCAA^−^) median levels of BCAA only, (**B,C**) hypertension (HT^+^) and BCAA levels, and (**D,E**) diabetes mellitus (DM^+^) and BCAA levels.

During a median follow-up of 17 years, 1235 participants died (41.2% of cohort; median age 85.8 years [IQR 81.7–89.7]; 58.0% male). 972 deaths (78.7% of total) were non-cardiovascular (39.4% infection, 38.6% cancer or neoplasia, and 22.0% miscellaneous) (Table S2), and 263 (21.3% of total) were attributable to ischaemic heart disease, stroke, and heart failure that comprised 81.7% of cardiovascular deaths (Table S1). Kaplan–Meier analysis revealed no statistically significant effect of BCAA levels on survival in women but a significant association with improved survival in men who had above-median levels of BCAA (*p* = 0.029) (Figure 2A). Intriguingly, BCAA levels did not significantly affect survival in participants with hypertension or diabetes but were for the most part significant in patients without hypertension or diabetes (HT^−^BCAA^high^ vs. HT^−^BCAA^low^, *p* = 0.0018 [male] and *p* = 0.054 [female]; DM^−^BCAA^high^ vs. DM^−^BCAA^low^, *p* = 0.0064 [male] and *p* = 0.042 [female]) (Figure 2B–E). Collectively, we observed that above-median levels of BCAA was associated with improved survival only in individuals without hypertension (Figure 2B,C) or diabetes (Figure 2D,E).

### 3.4. Mortality Hazard and Nonlinearity of BCAA Effects in Cardiometabolic Disease

Consistent with the results from Kaplan–Meier analysis, Cox regression models indicated that increased log(BCAA) was significantly associated with reduced all-cause mortality only in participants without hypertension (HT^−^; adjusted HR 0.43 [95% CI 0.25–0.75], *p* = 0.028) and diabetes (DM^−^; adjusted HR 0.39 [95% CI 0.25–0.61], *p* < 0.0001), whereas in participants with hypertension (HT^+^) or diabetes (DM^+^), no significant effect was observed (Table 3). BMI did not have a significant impact on all-cause mortality except in older adults with diabetes in whom increased BMI was associated with reduced mortality hazard (adjusted HR 0.93 [95% CI 0.88–0.97], *p* = 0.0025) (Table 3). Frailty and physical activity, assessed by the frailty index and PASE, respectively, had marginal or no effects. Active smoking and Egfr were major determinants of all-cause mortality (Table 3). The combination of HT and DM as a single variable was assessed and showed varying effects on adjusted Cox regression (Table S4).

**Figure 3:**
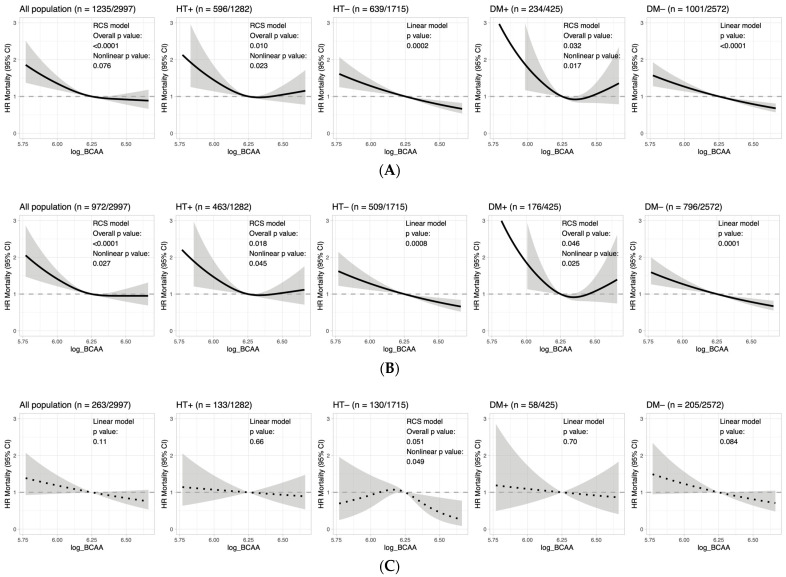
Cox regression modelling of BCAA levels on all-cause, non-cardiovascular and cardiovascular mortality. Shown are best-fitting restricted cubic spline or linear term models selected according to the lowest Aikake information criterion (AIC) values (see Table S4). Models were adjusted for age, sex, smoking, frailty index, eGFR, and the use of antihypertensive and hypoglycaemic drugs. HR, hazard ratio. ‘+’ and ‘−’ denote presence and absence, respectively, of the condition. (**A**) All-cause mortality. (**B**) Non-cardiovascular mortality. (**C**) Cardiovascular mortality.

To determine if nonlinearity could explain the heterogeneous results between subgroups seen in the linear models, Cox regression with restricted cubic spline models were used (Figure 3 and Figure S1, Tables S5 and S6). Of note, a U-shaped relationship was observed between BCAA levels and all-cause mortality for the best-fit models in participants with hypertension (HT^+^, nonlinear *p* = 0.023) or diabetes (DM^+^, nonlinear *p* = 0.017) (Figure 3A); whereas in those without hypertension or diabetes, the best-fit models were linear (HT^−^, linear *p* = 0.0002 and DM^−^, linear *p* < 0.0001) (Figure 3A). Further classification of mortality into cardiovascular and non-cardiovascular causes revealed that the nonlinear U-shaped relationship was primarily observed in the latter (Figure 3B,C). The restricted cubic spline models suggested that when fasting serum BCAA concentration fell outside the range of exp(6.2) to exp(6.3), or approximately 493–545 µmol/L, mortality hazard was increased, particularly at low concentrations (Figure 3A,B).

### 3.5. Serum BCAA Levels at Baseline and Year 14 Can Predict Future Mortality

We next compared fasting serum BCAA concentration at baseline (0Y, n = 2997) versus 14 years (14Y, n = 860) in four adjusted Cox regression models, and determined that increased BCAA levels were consistently and significantly associated with reduced incident all-cause death during a median follow-up of 17 years (0Y, *p* < 0.0001 to 0.0090, n = 2997; 14Y, *p* = 0.0047 to 0.0069, n = 860) (Table 4). Multivariable Cox regression using model 2 determined from baseline and 14Y that each 10% increase in BCAA level was associated with a reduction of 7% (*p* = 0.0002) and 16% (*p* = 0.0057), respectively, in mortality hazard. Independent predictors of increased mortality at both 0Y and 14Y time points in all four models were age and active smoking, whereas higher eGFR and log(BCAA) were protective (Table 4). Frailty at baseline may partially explain mortality hazard (*p* < 0.0001 to 0.0003, n = 2997). Combined variables comprising hypertension (HT) and/or antihypertensive drugs, or diabetes mellitus (DM) and/or hypoglycaemic drugs were also analysed (Table 5).

## 4. Discussion

This prospective study represents the first validated and longest time-to-event analysis of circulating BCAA levels as predictors of mortality in a cohort of ageing older adults. One of our main findings was that the impact of hypertension and diabetes on the relationship between circulating BCAA levels and non-cardiovascular mortality was nonlinear, cautioning the careful interpretation of data on survival and longevity in older adults, particularly the oldest-old. We provided validated evidence that BCAA levels could be informative for gauging future mortality risks among older adults. Advancing old age is associated with a catabolic state in which maintenance of energy homeostasis and physiological reserve impact survival and longevity. Indeed, recent studies have pointed to the negative impact of diabetes mellitus, hyperglycaemia, and/or hypertension on functional impairment and adverse outcomes in older age [38,39,40], which remain poorly understood. Intriguingly, we observed that the relationship between fasting serum BCAA concentration and mortality risk changed from a linear to U-shaped distribution in participants with hypertension and diabetes, suggesting that deviation from median levels may represent a form of failure to maintain BCAA and metabolic energy homeostasis (dyshomeostasis) in patients with cardiometabolic disease. 

Accumulating preclinical evidence points to the importance of BCAA homeostasis in health and lifespan [18]. While elevated BCAA levels are recognised risks for future cardiovascular disease and events [12,13,14,15,16,17], at least two studies in high-risk patients found higher BCAA levels to be independently predictive of mortality in the short and long term (median follow-up of 3.1 and 10.5 years, respectively) [41,42]. In the acute clinical setting of sepsis/septic shock [43] and in chronic kidney disease (mean eGFR 48 mL/min/1.73 m^2^) [44], increased BCAA levels were associated with reduced mortality hazard. Our results are in line with those findings that increased BCAA levels conferred survival advantage, and provided informative insight and prognostic power into the very long term through interval analysis at baseline (0Y) and 14Y to predict future mortality during a median follow-up of 17 years.

Available studies have found increased BCAA levels to be associated with cardiometabolic disorders, incident adverse cardiovascular disease and mortality in adults in their 50s and 60s [12,13,14,15,16,17,41,42]. Our study included participants who were well into their 70s at baseline and 80 s at 14Y (Table 2), with a median age of 85.8 years at the time of death, thus, representative of the oldest-old. There is a scarcity of information on circulating factors that influence survival in this population. However, there are also published data suggesting that low levels of BCAAs may be detrimental to survival in old age [13,26]. Le Couteur et al. reported that BCAA levels in the lowest quartile of a cohort of older Australian men were associated with increased mortality [26], supporting the premise that energy metabolism from sufficient nutritional and physiological reserve were crucial to longevity in older adults. In the oldest-old, increased BCAA levels could be interpreted as a negative consequence of cardiometabolic disease, or reflect sufficient physiological and energy reserve in those without hypertension or diabetes. On a deeper level, our findings suggested that hypertension and diabetes had abrogating effects on the potential benefits of higher BCAA levels in older adults that otherwise was associated with improved survival in those without hypertension and diabetes.

In translating knowledge into practice, clinicians may first determine if an older adult has hypertension and/or diabetes, then compare levels of fasting serum BCAA concentrations relative to population median levels to gauge health status. Whereas excessively high or low BCAA levels may indicate increased mortality hazard in older adults with hypertension and/or diabetes, individuals without cardiometabolic disease in whom low BCAA levels are detected may benefit from a detailed evaluation and review of potentially reversible ageing-related comorbidities (e.g., chronic kidney disease), malnutrition, and frailty. Further studies are needed to determine if the guideline-directed management of hypertension and diabetes in conjunction with the modulation of BCAA levels can restore metabolic homeostasis and further improve outcomes. Recently, a randomised placebo-controlled clinical trial of sodium phenylbutyrate, an accelerator of BCAA metabolism, demonstrated successful proof-of-concept and safety in human patients with type 2 diabetes mellitus [45]. While experimental modulation of circulating amino acid level (e.g., glutamine) has shown promise in improving glucose tolerance and blood pressure via the alteration of amino acid balance (e.g., glutamine-to-glutamate ratio) in rodents [22], further clinical trials on the safety and efficacy of this therapeutic approach are awaited.

This study benefits from several strengths, including a very long time-to-event analysis with a median follow-up time of 17 years in a well-characterised older adult population of both men and women. No previous study has provided a comparably long follow-up with an additional time point (14Y) for the validation of mortality prediction. There are, however, several limitations. First, selection bias is possible due to the recruitment of community-living older adults who were ambulatory. Highly frail, debilitated and institutionalised older adults with advanced multimorbidity and disabilities were not represented by this study population. Second, sampling bias might have been introduced at 14Y follow-up, as relatively healthier and less debilitated people were more likely to return for follow-up assessment. This could have been avoided if home visits and assessments were performed. However, this was beyond the capacity of the research team. Third, our study relied on the Deaths Registries and review of medical records to determine the causes of death. It is possible that the accuracy of determination of deaths can vary depending on the certifying clinician and the clinical circumstance. Fourth, the proportion of cardiovascular deaths in this cohort may be considered relatively low (21%) compared with that of mainland China (40%) [46,47] and the global population (32%) [48,49]. This could be explained by differences in subethnic, genetic and environmental determinants, including diet, lifestyle and access to healthcare and health systems that differed between populations [50]. Finally, the analysis performed in this study did not account for latent variables and other factors, including blood lipid profile [25,51,52] and gut microbiota [53], that could have substantive influence on the precision of effect size and risk estimation.

In conclusion, this study provided further insight into the complex interrelationship between BCAAs and mortality hazard in old age that may be powerfully modulated by hypertension and/or diabetes. Our validated findings increase the robustness of results, and have implications for the management and care planning of older adults. Further studies are needed to determine if the guided modulation of BCAA levels is associated with survival benefits.

## Figures and Tables

**Table 1:** Baseline characteristics of study participants stratified by hypertension (HT) and diabetes mellitus (DM) status.

Characteristic	Overall	HT	No HT	*p* Value	DM	No DM	*p* Value
N	2997 (100%)	1282 (42.8%)	1715 (57.2%)	-	425 (14.2%)	2572 (85.8%)	-
Age, years	71 (68–75)	72 (68–75)	71 (68–75)	<0.0001	71 (68–76)	71 (68–75)	0.11
Sex, male	1424 (47.5%)	594 (46.3%)	830 (48.4%)	0.26	206 (48.5%)	1218 (47.3%)	0.67
Body weight, kg	58 (51.9–65)	59.8 (53.6–66.5)	56.7 (50.8–63.7)	<0.0001	59.9 (53.9–66.8)	57.7 (51.6–64.5)	<0.0001
BMI, kg/m^2^	23.6 (21.7–25.8)	24.4 (22.4–26.4)	23 (21.2–25.3)	<0.0001	24.3 (22.4–26.4)	23.5 (21.5–25.6)	<0.0001
eGFR, mL/min/1.73 m^2^	90 (77.8–95.4)	86.8 (73.2–94.4)	91.7 (81.6–96)	<0.0001	89.6 (75.8–95.8)	90.1 (78.1–95.3)	0.52
Frailty index	0.11 (0.04–0.15)	0.13 (0.11–0.19)	0.06 (0.04–0.11)	<0.0001	0.17 (0.11–0.21)	0.09 (0.04–0.13)	<0.0001
PASE	85.6 (65.3–112.6)	84.7 (62.2–110.4)	86.3 (67.6–115.3)	0.0016	85.4 (65.4–111.3)	85.6 (65.3–112.6)	0.94
Smoking							
Never	1979 (66%)	868 (67.7%)	1111 (64.7%)	0.094	282 (66.4%)	1697 (66%)	0.88
Past	840 (28%)	371 (28.9%)	469 (27.3%)	0.34	125 (29.4%)	715 (27.8%)	0.49
Current	178 (5.9%)	43 (3.4%)	135 (7.9%)	<0.0001	18 (4.2%)	160 (6.2%)	0.11
Comorbidities							
Myocardial infarction	277 (9.2%)	172 (13.4%)	105 (6.1%)	<0.0001	63 (14.8%)	214 (8.3%)	<0.0001
Angina pectoris	241 (8.0%)	139 (10.8%)	102 (6.0%)	<0.0001	45 (10.6%)	196 (7.6%)	0.037
Stroke	117 (3.9%)	82 (6.4%)	35 (2.0%)	<0.0001	25 (5.9%)	92 (3.6%)	0.023
Heart failure	115 (3.8%)	73 (5.7%)	42 (2.4%)	<0.0001	25 (5.9%)	90 (3.5%)	0.018
COPD	218 (7.3%)	87 (6.8%)	131 (7.6%)	0.37	26 (6.1%)	192 (7.5%)	0.32
CKD (eGFR < 60)	189 (6.3%)	127 (9.9%)	62 (3.6%)	<0.0001	28 (6.6%)	161 (6.3%)	0.80
Cardiovascular drugs							
CCB	548 (18.3%)	509 (39.7%)	39 (2.3%)	<0.0001	131 (30.8%)	417 (16.2%)	<0.0001
β-blocker	489 (16.3%)	430 (33.5%)	59 (3.4%)	<0.0001	108 (25.4%)	381 (14.8%)	<0.0001
ACEI/ARB	342 (1.4%)	314 (24.5%)	28 (1.6%)	<0.0001	111 (26.1%)	231 (9.0%)	<0.0001
Aspirin	322 (10.7%)	216 (16.8%)	106 (6.2%)	<0.0001	85 (20.0%)	237 (9.2%)	<0.0001
Diuretic	293 (9.8%)	267 (20.8%)	26 (1.5%)	<0.0001	56 (13.2%)	237 (9.2%)	0.011
Statin	188 (6.3%)	133 (10.4%)	55 (3.2%)	<0.0001	49 (11.5%)	139 (5.4%)	<0.0001
α-blocker	184 (6.1%)	110 (8.6%)	74 (4.3%)	<0.0001	31 (7.3%)	153 (5.9%)	0.29
Nitrate	182 (6.1%)	125 (9.8%)	57 (3.3%)	<0.0001	43 (10.1%)	139 (5.4%)	0.0002
Digoxin	16 (0.5%)	5 (0.4%)	11 (0.6%)	0.35	1 (0.2%)	15 (0.6%)	0.36
Warfarin	12 (0.4%)	5 (0.4%)	7 (0.4%)	0.94	4 (0.9%)	8 (0.3%)	0.57
Antihypertensive drugs *	1177 (39.3%)	990 (77.2%)	187 (10.9%)	<0.0001	258 (60.7%)	919 (35.7%)	<0.0001
Hypoglycaemic drugs	319 (10.6%)	223 (17.4%)	96 (5.6%)	<0.0001	311 (73.2%)	8 (0.3%)	<0.0001
Metformin	98 (3.3%)	67 (5.2%)	31 (1.8%)	<0.0001	97 (22.8%)	1 (0.04%)	<0.0001
Glibenclamide	61 (2.0%)	38 (3.0%)	23 (1.3%)	0.0019	61 (14.4%)	0 (0%)	<0.0001
Gliclazide	86 (2.9%)	58 (4.5%)	28 (1.6%)	<0.0001	83 (19.5%)	3 (0.1%)	<0.0001
Glipizide	16 (0.5%)	11 (0.9%)	5 (0.3%)	0.035	16 (3.8%)	0 (0%)	<0.0001
Acarbose	5 (0.2%)	5 (0.4%)	0 (0%)	0.0097	5 (1.2%)	0 (0%)	<0.0001
Insulin	3 (0.1%)	3 (0.2%)	0 (0%)	0.045	3 (0.7%)	0 (0%)	<0.0001
Tolbutamide	2 (0.1%)	2 (0.2%)	0 (0%)	0.102	2 (0.5%)	0 (0%)	0.0005

Median (interquartile range) value or count (percentage) is shown. BMI, body mass index. COPD, chronic obstructive pulmonary disease. CKD, chronic kidney disease. DM, diabetes mellitus. eGFR, estimated glomerular filtration rate by CKD-EPI formula. HT, hypertension. IQR, interquartile range. PASE, Physical Activity Scale for the Elderly. * Antihypertensive drugs include angiotensin-converting enzyme inhibitor (ACEI), angiotensin receptor blocker (ARB), calcium channel blocker (CCB), and α-blocker.

**Table 2:** Fasting serum BCAA concentrations measured at baseline (0Y) and at 14 years (14Y).

**0Y**							
**Variable**	**Overall** **(n = 2997)**	**HT** **(n = 1282)**	**No HT** **(n = 1715)**	***p* Value**	**DM** **(n = 425)**	**No DM** **(n = 2572)**	***p* Value**
Age, years	71 (68–75)	72 (68–75)	71 (68–75)	<0.0001	71 (68–76)	71 (68–75)	0.11
Sex, male	1424 (47.5%)	594 (46.3%)	830 (48.4%)	0.26	206 (48.5%)	1218 (47.3%)	0.67
Serum creatinine, µmol/L	70.3 (58.2–84.3)	72.8 (60.7–87.8)	68.4 (57.1–81.2)	<0.0001	70.7 (59.1–84.5)	70.3 (58.2–84.3)	0.66
Total BCAA, µmol/L	514.3 (463.2–574.6)	525.3 (474.6–592.8)	507 (455–560.9)	<0.0001	558.6 (501.9–616.1)	509 (457.7–565.5)	<0.0001
Isoleucine, µmol/L	78.9 (69.6–89.3)	80.3 (72.1–92.4)	77.2 (68.2–87.0)	<0.0001	86.1 (76.3–98.6)	77.7 (68.7–87.5)	<0.0001
Leucine, µmol/L	146.9 (131.3–164.2)	150 (134.9–169.1)	144.5 (129.0–160.9)	<0.0001	159.8 (143.4–177.8)	144.9 (129.5–161.7)	<0.0001
Valine, µmol/L	288.9 (259.2–323.0)	295.2 (265.0–331.1)	284.8 (255.7–316.1)	<0.0001	310.7 (283.0–340.4)	285.6 (256.5–318.9)	<0.0001
**14Y**							
**Variable**	**Overall** **(n = 860)**	**HT at 0Y** **(n = 339)**	**No HT at 0Y** **(n = 521)**	***p*** **value**	**DM at 0Y** **(n = 104)**	**No DM at 0Y** **(n = 756)**	***p*** **value**
Age, years	83 (81–86)	84 (81–87)	83 (80–86)	0.0005	83 (81–85)	83 (81–86)	0.94
Sex, male	408 (47.4%)	165 (48.7%)	243 (46.6%)	0.56	50 (48.1%)	358 (47.4%)	0.89
Serum creatinine, µmol/L	74.3 (63.4–87.5)	78.2 (65.8–93.9)	71.5 (62.0–83.6)	<0.0001	81 (68.0–98.2)	73 (62.8–85.7)	<0.0001
Total BCAA, µmol/L	421.6 (379.0–470.5)	433.5 (392.5–477.9)	413 (370.2–466.7)	0.0002	469.1 (406.1–507.4)	417.8 (374.9–464.7)	<0.0001
Isoleucine, µmol/L	53.6 (45.3–63.0)	56.8 (48.5–64.5)	51.8 (44.3–60.9)	<0.0001	62.8 (52.5–71.8)	52.9 (44.7–61.5)	<0.0001
Leucine, µmol/L	126.4 (112.5–141.4)	130.3 (115.9–143.9)	123.6 (110.0–139.6)	0.0002	140.1 (125.9–152.6)	124.4 (111.4–139.3)	<0.0001
Valine, µmol/L	240.2 (219.1–268.0)	243.3 (225.8–273.5)	237.3 (213.9–263.9)	0.0029	257.1 (231.3–281.1)	239 (215.8–265.1)	<0.0001

Median (interquartile range) value or count (percentage) is shown. BCAA, branched-chain amino acid. DM, diabetes mellitus. HT, hypertension.

**Table 3:** Multivariable Cox modelling of baseline independent variables on incident all-cause mortality stratified by hypertension (HT) and diabetes mellitus (DM) with adjustment for age, sex, BMI, smoking, frailty, Egfr, and use of antihypertensive and/or hypoglycaemic drugs.

Variable	HT^+^ (Deaths, n = 596/1282)		HT^−^ (Deaths, n = 639/1715)		DM^+^ (Deaths, n = 234/425)		DM^−^ (Deaths, n = 1001/2572)	
	HR (95% CI)	*p* Value	HR (95% CI)	*p* Value	HR (95% CI)	*p* Value	HR (95% CI)	*p* Value
Age	1.09 (1.07–1.11)	<0.0001	1.11 (1.09–1.13)	<0.0001	1.1 (1.07–1.13)	<0.0001	1.1 (1.09–1.11)	<0.0001
Sex	1.44 (1.16–1.78)	0.0008	1.66 (1.35–2.04)	<0.0001	1.41 (1.01–1.98)	0.043	1.62 (1.37–1.91)	<0.0001
BMI	1 (0.97–1.03)	0.88	0.98 (0.95–1)	0.085	0.93 (0.88–0.97)	0.0025	1 (0.98–1.02)	0.97
Previous smoking	1.32 (1.07–1.62)	0.0086	1.29 (1.06–1.57)	0.011	1.17 (0.84–1.63)	0.34	1.34 (1.14–1.57)	0.0003
Active smoking	2.4 (1.65–3.49)	<0.0001	1.85 (1.4–2.45)	<0.0001	1.99 (1.07–3.68)	0.029	2.09 (1.65–2.65)	<0.0001
Frailty index	3.43 (0.97–12.16)	0.057	3.69 (0.89–15.3)	0.072	3 (0.39–22.84)	0.29	3.5 (1.21–10.11)	0.021
PASE	1 (1–1)	0.31	1 (1–1)	0.21	1 (0.99–1)	0.096	1 (1–1)	0.18
Egfr	0.99 (0.98–0.99)	<0.0001	0.99 (0.99–1)	0.014	0.99 (0.98–1)	0.0052	0.99 (0.98–0.99)	<0.0001
log(BCAA) ^‡^	0.55 (0.3–1.02)	0.057	0.43 (0.25–0.75)	0.0029	0.98 (0.37–2.58)	0.97	0.39 (0.25–0.61)	<0.0001
Antihypertensive drugs *	1.12 (0.91–1.38)	0.29	1.3 (1.02–1.66)	0.033	1.15 (0.84–1.57)	0.39	1.2 (1.04–1.39)	0.015
Hypoglycaemic drugs ^†^	1.69 (1.36–2.08)	<0.0001	1.61 (1.17–2.22)	0.0039	1.13 (0.83–1.54)	0.45	1.73 (0.77–3.9)	0.18

BCAA, branched-chain amino acid. BMI, body mass index. DM, diabetes mellitus. Egfr, estimated glomerular filtration rate by CKD-EPI formula. HR, hazard ratio. HT, hypertension. PASE, Physical Activity Scale for the Elderly. * Antihypertensive drugs include α-blocker, angiotensin-converting enzyme inhibitor/angiotensin receptor blocker, β-blocker, calcium channel blocker, diuretics and nitrate. ^†^ Hypoglycaemic drugs include metformin, glibenclamide, gliclazide, glipizide, acarbose, insulin, and tolbutamide. ^‡^ HR represents 1 unit increase in log scale which is approximately equivalent to 2.718 times increase in BCAA level. Deaths (n) among total number of study participants in subgroup within parentheses. See also Methods and Table 1.

**Table 4:** Multiple Cox regression showing association between fasting serum BCAA concentration and incident all-cause mortality at 0Y and 14Y *.

Variable	Model 1 ^a^		Model 2 ^b^		Model 3 ^c^		Model 4 ^d^	
	HR (95% CI)	*p* Value	HR (95% CI)	*p* Value	HR (95% CI)	*p* Value	HR (95% CI)	*p* Value
***Baseline (0Y)***								
Age	1.10 (1.09–1.11)	<0.0001	1.10 (1.09–1.11)	<0.0001	1.10 (1.09–1.11)	<0.0001	1.10 (1.09–1.11)	<0.0001
Sex	1.56 (1.35–1.80)	<0.0001	1.55 (1.34–1.79)	<0.0001	1.52 (1.32–1.76)	<0.0001	1.57 (1.36–1.81)	<0.0001
BMI	0.99 (0.97–1.01)	0.31	0.99 (0.97–1.01)	0.21	0.99 (0.97–1.01)	0.25	0.99 (0.97–1.01)	0.32
Previous smoking	1.30 (1.13–1.49)	0.0003	1.30 (1.13–1.50)	0.0003	1.30 (1.13–1.50)	0.0003	1.31 (1.13–1.50)	0.0002
Active smoking	1.98 (1.59–2.47)	<0.0001	2.05 (1.64–2.56)	<0.0001	2.04 (1.64–2.54)	<0.0001	2.01 (1.61–2.51)	<0.0001
Frailty index	14.48 (6.79–30.89)	<0.0001	5.28 (2.16–12.92)	0.0003	7.95 (3.31–19.12)	<0.0001	6.99 (3.12–15.66)	<0.0001
eGFR	0.99 (0.99–0.99)	<0.0001	0.99 (0.98–0.99)	<0.0001	0.99 (0.99–0.99)	<0.0001	0.99 (0.98–0.99)	<0.0001
log(BCAA) ^†^	0.58 (0.39–0.87)	0.0090	0.47 (0.31–0.70)	0.0002	0.58 (0.39–0.87)	0.0078	0.47 (0.32–0.71)	0.0003
HT	-	-	1.08 (0.95–1.24)	0.23	-	-	-	-
DM	-	-	1.61 (1.38–1.89)	<0.0001	-	-	-	-
Antihypertensive drugs	-	-	-	-	1.21 (1.06–1.38)	0.0056	-	-
Hypoglycaemic drugs	-	-	-	-	-	-	1.67 (1.4–1.99)	<0.0001
***14Y***								
Age	1.10 (1.04–1.15)	0.0002	1.10 (1.04–1.15)	0.0002	1.10 (1.05–1.15)	0.0002	1.10 (1.04–1.15)	0.0002
Sex	1.70 (1.03–2.79)	0.037	1.62 (0.98–2.67)	0.058	1.61 (0.98–2.65)	0.061	1.69 (1.03–2.78)	0.038
BMI (baseline)	0.95 (0.88–1.02)	0.15	0.95 (0.88–1.02)	0.14	0.95 (0.88–1.02)	0.19	0.95 (0.88–1.02)	0.15
Previous smoking (baseline)	1.22 (0.74–2.00)	0.43	1.25 (0.76–2.05)	0.38	1.22 (0.75–2.01)	0.42	1.23 (0.75–2.01)	0.42
Active smoking (baseline)	2.46 (1.12–5.38)	0.025	2.67 (1.21–5.91)	0.015	2.68 (1.21–5.90)	0.015	2.45 (1.12–5.37)	0.025
Frailty index (baseline)	8.42 (0.37–189.70)	0.18	2.56 (0.06–105.63)	0.62	1.71 (0.04–69.51)	0.78	7.52 (0.29–196.15)	0.23
eGFR	0.97 (0.96–0.99)	<0.0001	0.98 (0.96–0.99)	0.0002	0.98 (0.96–0.99)	0.0001	0.97 (0.96–0.99)	0.0001
log(BCAA) ^†^	0.16 (0.04–0.61)	0.0069	0.15 (0.04–0.58)	0.0057	0.15 (0.04–0.56)	0.0047	0.16 (0.04–0.60)	0.0067
HT (baseline)	-	-	1.41 (0.88–2.25)	0.15	-	-	-	-
DM (baseline)	-	-	1.01 (0.53–1.93)	0.97	-	-	-	-
Antihypertensive drugs (baseline)	-	-	-	-	1.51 (0.94–2.43)	0.092	-	-
Hypoglycaemic drugs (baseline)	-	-	-	-	-	-	1.09 (0.55–2.16)	0.81

BCAA, branched-chain amino acid. BMI, body mass index. DM, diabetes mellitus. eGFR, estimated glomerular filtration rate. HR, hazard ratio. HT, hypertension. * Of 2997 and 860 available participants included in the baseline and 14Y analysis, 1235 and 100 died, respectively, from all causes. A lack of significant effects of HT and DM in Model 2 may be explained by nonlinear interrelationships illustrated in Figure 3. ^a^ Model 1 = log(BCAA) as the independent variable, and adjusted for age, sex, BMI, frailty, smoking status (previous and active smoking), frailty index and eGFR. ^b^ Model 2 = Model 1 additionally adjusted for HT and DM status. ^c^ Model 3 = Model 1 additionally adjusted for the use of antihypertensive drugs including α-blocker, angiotensin-converting enzyme inhibitor/angiotensin receptor blocker, β-blocker, calcium channel blocker, diuretics and nitrate. ^d^ Model 4 = Model 1 additionally adjusted for the use of hypoglycaemic drugs including metformin, glibenclamide, gliclazide, glipizide, acarbose, insulin, and tolbutamide. ^†^ Each 1 unit increase in HR on log scale is approximately equivalent to 2.718 times increase in BCAA level. See also Methods and Table 1 for details.

**Table 5:** Association between fasting serum BCAA concentration and incident all-cause mortality at 0Y and 14Y using combined variables comprising hypertension (HT) and/or antihypertensive drugs, or diabetes mellitus (DM) and/or hypoglycaemic drugs *.

Variable	Model 1 ^a^		Model 2 ^b^		Model 3 ^c^	
	HR (95% CI)	*p* Value	HR (95% CI)	*p* Value	HR (95% CI)	*p* Value
***Baseline (0Y)***						
Age	1.10 (1.09–1.11)	<0.0001	1.1 (1.09–1.11)	<0.0001	1.1 (1.09–1.11)	<0.0001
Sex	1.56 (1.35–1.80)	<0.0001	1.56 (1.35–1.8)	<0.0001	1.53 (1.32–1.76)	<0.0001
BMI	0.99 (0.97–1.01)	0.31	0.99 (0.97–1.01)	0.27	0.99 (0.97–1.01)	0.19
Previous smoking	1.30 (1.13–1.49)	0.0003	1.3 (1.13–1.5)	0.0003	1.31 (1.14–1.51)	0.0002
Active smoking	1.98 (1.59–2.47)	<0.0001	2.03 (1.63–2.53)	<0.0001	2.09 (1.68–2.61)	<0.0001
Frailty index	14.48 (6.79–30.89)	<0.0001	5.34 (2.19–13.05)	0.0002	3.86 (1.53–9.75)	0.0043
eGFR	0.99 (0.99–0.99)	<0.0001	0.99 (0.98–0.99)	<0.0001	0.99 (0.99–0.99)	<0.0001
log(BCAA)^†^	0.58 (0.39–0.87)	0.0090	0.47 (0.31–0.7)	0.0003	0.46 (0.31–0.7)	0.0002
HT and antihypertensive drugs	-	-	1.12 (0.98–1.28)	0.10	-	-
DM and hypoglycaemic drugs	-	-	1.65 (1.38–1.96)	<0.0001	-	-
HT or antihypertensive drugs	-	-	-	-	1.17 (1.02–1.34)	0.024
DM or hypoglycaemic drugs	-	-	-	-	1.62 (1.39–1.9)	<0.0001
***14Y***						
Age	1.10 (1.04–1.15)	0.0002	1.1 (1.05–1.15)	0.0002	1.1 (1.04–1.15)	0.0002
Sex	1.70 (1.03–2.79)	0.037	1.63 (0.99–2.68)	0.053	1.61 (0.97–2.66)	0.065
BMI (baseline)	0.95 (0.88–1.02)	0.15	0.95 (0.88–1.02)	0.18	0.95 (0.88–1.02)	0.15
Previous smoking (baseline)	1.22 (0.74–2.00)	0.43	1.26 (0.77–2.06)	0.37	1.23 (0.75–2.02)	0.42
Active smoking (baseline)	2.46 (1.12–5.38)	0.025	2.67 (1.21–5.87)	0.015	2.67 (1.2–5.92)	0.016
Frailty index (baseline)	8.42 (0.37–189.70)	0.18	1.61 (0.04–65.75)	0.80	2.5 (0.05–120.87)	0.65
eGFR	0.97 (0.96–0.99)	<0.0001	0.98 (0.96–0.99)	0.0002	0.97 (0.96–0.99)	0.0001
log(BCAA) ^†^	0.16 (0.04–0.61)	0.0069	0.15 (0.04–0.55)	0.0045	0.15 (0.04–0.58)	0.0056
HT and antihypertensive drugs (baseline)	-	-	1.6 (1–2.55)	0.049	-	-
DM and hypoglycaemic drugs (baseline)	-	-	1.06 (0.53–2.11)	0.87	-	-
HT or antihypertensive drugs (baseline)	-	-	-	-	1.35 (0.83–2.21)	0.21
DM or hypoglycaemic drugs (baseline)	-	-	-	-	1.01 (0.53–1.92)	0.97

BCAA, branched-chain amino acid. BMI, body mass index. DM, diabetes mellitus. eGFR, estimated glomerular filtration rate. HR, hazard ratio. HT, hypertension.* Of 2997 and 860 available participants included in the 0Y and 14Y analysis, 1235 and 100 died, respectively, from all causes. ^a^ Model 1 = log(BCAA) as the independent variable, and adjusted for age, sex, BMI, frailty, smoking status (previous and active smoking), frailty index and eGFR. ^b^ Model 2 = Model 1 + additionally adjusted for combined variables, (HT and antihypertensive drugs) and (DM and hypoglycaemic drugs). ^c^ Model 3 = Model 1 + additionally adjusted for combined variables, (HT or antihypertensive drugs) and (DM or hypoglycaemic drugs). ^†^ Each 1 unit increase in HR on log scale is approximately equivalent to 2.718 times increase in BCAA level.

## Data Availability

The data that support the findings of this study are available from the corresponding author upon reasonable request.

## Supplementary Materials

The following supporting information can be downloaded at: https://www.mdpi.com/article/10.3390/biom13081252/s1, Figure S1: Restricted cubic splines of log(BCAA) modelled on all-cause, non-cardiovascular and cardiovascular mortality in older adults stratified by the combined status of hypertension (HT) and diabetes mellitus (DM); Table S1: Cardiovascular deaths classified by ICD-10 codes among 2997 older adults during a median follow-up of 17 years; Table S2: Non-cardiovascular deaths classified by ICD-10 codes among 2997 older adults during a median follow- up of 17 years; Table S3: Serum branched-chain amino acid levels were consistently higher in older men than women at both time points, 0Y and 14Y; Table S4: Adjusted Cox regression modelling of baseline independent variables on incident all-cause mortality stratified by the combined status of hypertension (HT) and diabetes (DM) with adjustment for age, sex, BMI, smoking, frailty, eGFR, and use of antihypertensive and/or hypoglycaemic drugs; Table S5: Details pertinent to the selection of best fitting models from restricted cubic spline and adjusted Cox regression of BCAA on all-cause, non-cardiovascular and cardiovascular mortality in older adults stratified by hypertension (HT) or diabetes (DM) status; Table S6: Details pertinent to the selection of best fitting models from restricted cubic spline and adjusted Cox regression of BCAA on all-cause, non-cardiovascular and cardiovascular mortality in older adults stratified by the combined status of hypertension (HT) and diabetes mellitus (DM).

## References

[1] United Nations. https://www.un.org/en/global-issues/ageing.

[2] GBD 2019 Diseases and Injuries Collaborators (2020). Global burden of 369 diseases and injuries in 204 countries and territories, 1990–2019: A systematic analysis for the Global Burden of Disease Study 2019. Lancet.

[3] Hambleton I.R., Caixeta R., Jeyaseelan S.M., Luciani S., Hennis A.J.M. (2023). The rising burden of non-communicable diseases in the Americas and the impact of population aging: A secondary analysis of available data. Lancet Reg. Health Am..

[4] Ford E.S. (2011). Trends in mortality from all causes and cardiovascular disease among hypertensive and nonhypertensive adults in the United States. Circulation.

[5] Rapsomaniki E., Timmis A., George J., Pujades-Rodriguez M., Shah A.D., Denaxas S., White I.R., Caulfield M.J., Deanfield J.E., Smeeth L. (2014). Blood pressure and incidence of twelve cardiovascular diseases: Lifetime risks, healthy life-years lost, and age-specific associations in 1.25 million people. Lancet.

[6] Bozkurt B., Aguilar D., Deswal A., Dunbar S.B., Francis G.S., Horwich T., Jessup M., Kosiborod M., Pritchett A.M., Ramasubbu K. (2016). Contributory Risk and Management of Comorbidities of Hypertension, Obesity, Diabetes Mellitus, Hyperlipidemia, and Metabolic Syndrome in Chronic Heart Failure: A Scientific Statement From the American Heart Association. Circulation.

[7] Sun H., Saeedi P., Karuranga S., Pinkepank M., Ogurtsova K., Duncan B.B., Stein C., Basit A., Chan J.C.N., Mbanya J.C. (2021). IDF diabetes Atlas: Global, regional and country-level diabetes prevalence estimates for 2021 and projections for 2045. Diabetes Res. Clin. Pract..

[8] NCD Risk Factor Collaboration (NCD-RisC) (2021). Worldwide trends in hypertension prevalence and progress in treatment and control from 1990 to 2019: A pooled analysis of 1201 population-representative studies with 104 million participants. Lancet.

[9] McGarrah R.W., White P.J. (2023). Branched-chain amino acids in cardiovascular disease. Nat. Rev. Cardiol..

[10] Lynch C.J., Adams S.H. (2014). Branched-chain amino acids in metabolic signalling and insulin resistance. Nat. Rev. Endocrinol..

[11] Zhang Z.Y., Monleon D., Verhamme P., Staessen J.A. (2018). Branched-Chain Amino Acids as Critical Switches in Health and Disease. Hypertension.

[12] Flores-Guerrero J.L., Groothof D., Connelly M.A., Otvos J.D., Bakker S.J.L., Dullaart R.P.F. (2019). Concentration of Branched-Chain Amino Acids Is a Strong Risk Marker for Incident Hypertension. Hypertension.

[13] Sun H., Olson K.C., Gao C., Prosdocimo D.A., Zhou M., Wang Z., Jeyaraj D., Youn J.Y., Ren S., Liu Y. (2016). Catabolic Defect of Branched-Chain Amino Acids Promotes Heart Failure. Circulation.

[14] Lim L.L., Lau E.S.H., Fung E., Lee H.M., Ma R.C.W., Tam C.H.T., Wong W.K.K., Ng A.C.W., Chow E., Luk A.O.Y. (2020). Circulating branched-chain amino acids and incident heart failure in type 2 diabetes: The Hong Kong Diabetes Register. Diabetes Metab. Res. Rev..

[15] Du X., Li Y., Wang Y., You H., Hui P., Zheng Y., Du J. (2018). Increased branched-chain amino acid levels are associated with long-term adverse cardiovascular events in patients with STEMI and acute heart failure. Life Sci..

[16] Du X., You H., Li Y., Wang Y., Hui P., Qiao B., Lu J., Zhang W., Zhou S., Zheng Y. (2018). Relationships between circulating branched chain amino acid concentrations and risk of adverse cardiovascular events in patients with STEMI treated with PCI. Sci. Rep..

[17] Tobias D.K., Lawler P.R., Harada P.H., Demler O.V., Ridker P.M., Manson J.E., Cheng S., Mora S. (2018). Circulating Branched-Chain Amino Acids and Incident Cardiovascular Disease in a Prospective Cohort of US Women. Circ. Genom. Precis. Med..

[18] Solon-Biet S.M., Cogger V.C., Pulpitel T., Wahl D., Clark X., Bagley E., Gregoriou G.C., Senior A.M., Wang Q.P., Brandon A.E. (2019). Branched chain amino acids impact health and lifespan indirectly via amino acid balance and appetite control. Nat. Metab..

[19] Zhang Z.Y., Marrachelli V.G., Yang W.Y., Trenson S., Huang Q.F., Wei F.F., Thijs L., Van Keer J., Monleon D., Verhamme P. (2019). Diastolic left ventricular function in relation to circulating metabolic biomarkers in a population study. Eur. J. Prev. Cardiol..

[20] Tanada Y., Shioi T., Kato T., Kawamoto A., Okuda J., Kimura T. (2015). Branched-chain amino acids ameliorate heart failure with cardiac cachexia in rats. Life Sci..

[21] D’Antona G., Ragni M., Cardile A., Tedesco L., Dossena M., Bruttini F., Caliaro F., Corsetti G., Bottinelli R., Carruba M.O. (2010). Branched-chain amino acid supplementation promotes survival and supports cardiac and skeletal muscle mitochondrial biogenesis in middle-aged mice. Cell Metab..

[22] Cheng S., Rhee E.P., Larson M.G., Lewis G.D., McCabe E.L., Shen D., Palma M.J., Roberts L.D., Dejam A., Souza A.L. (2012). Metabolite profiling identifies pathways associated with metabolic risk in humans. Circulation.

[23] Magnusson M., Lewis G.D., Ericson U., Orho-Melander M., Hedblad B., Engström G., Ostling G., Clish C., Wang T.J., Gerszten R.E. (2013). A diabetes-predictive amino acid score and future cardiovascular disease. Eur. Heart J..

[24] Sun L., Hu C., Yang R., Lv Y., Yuan H., Liang Q., He B., Pang G., Jiang M., Dong J. (2017). Association of circulating branched-chain amino acids with cardiometabolic traits differs between adults and the oldest-old. Oncotarget.

[25] Hamaya R., Mora S., Lawler P.R., Cook N.R., Ridker P.M., Buring J.E., Lee I.M., Manson J.E., Tobias D.K. (2021). Association of Plasma Branched-Chain Amino Acid With Biomarkers of Inflammation and Lipid Metabolism in Women. Circ. Genom. Precis. Med..

[26] Le Couteur D.G., Ribeiro R., Senior A., Hsu B., Hirani V., Blyth F.M., Waite L.M., Simpson S.J., Naganathan V., Cumming R.G. (2020). Branched Chain Amino Acids, Cardiometabolic Risk Factors and Outcomes in Older Men: The Concord Health and Ageing in Men Project. J. Gerontol. A Biol. Sci. Med. Sci..

[27] Ni M.Y., Canudas-Romo V., Shi J., Flores F.P., Chow M.S.C., Yao X.I., Ho S.Y., Lam T.H., Schooling C.M., Lopez A.D. (2021). Understanding longevity in Hong Kong: A comparative study with long-living, high-income countries. Lancet Public Health.

[28] Woo J., Leung J.C., Wong S.Y. (2010). Impact of childhood experience of famine on late life health. J. Nutr. Health Aging.

[29] Kin C.F., Shan W.S., Shun L.J., Chung L.P., Jean W. (2007). Experience of famine and bone health in post-menopausal women. Int. J. Epidemiol..

[30] Centre for Health Protection (2023). Hypertension.

[31] World Health Organization (2020). HEARTS D: Diagnosis and Management of Type 2 Diabetes (HEARTS-D).

[32] Rockwood K., Andrew M., Mitnitski A. (2007). A comparison of two approaches to measuring frailty in elderly people. J. Gerontol. Ser. A Biol. Sci. Med. Sci..

[33] Levey A.S., Stevens L.A., Schmid C.H., Zhang Y.L., Castro A.F., Feldman H.I., Kusek J.W., Eggers P., Van Lente F., Greene T. (2009). A new equation to estimate glomerular filtration rate. Ann. Intern. Med..

[34] Su Y., Elshorbagy A., Turner C., Refsum H., Chan R., Kwok T. (2019). Circulating amino acids are associated with bone mineral density decline and ten-year major osteoporotic fracture risk in older community-dwelling adults. Bone.

[35] Soininen P., Kangas A.J., Würtz P., Suna T., Ala-Korpela M. (2015). Quantitative serum nuclear magnetic resonance metabolomics in cardiovascular epidemiology and genetics. Circ. Cardiovasc. Genet..

[36] Bhinderwala F., Wase N., DiRusso C., Powers R. (2018). Combining Mass Spectrometry and NMR Improves Metabolite Detection and Annotation. J. Proteome Res..

[37] Ritchie S.C., Surendran P., Karthikeyan S., Lambert S.A., Bolton T., Pennells L., Danesh J., Di Angelantonio E., Butterworth A.S., Inouye M. (2023). Quality control and removal of technical variation of NMR metabolic biomarker data in ~120,000 UK Biobank participants. Sci. Data.

[38] Pansini A., Lombardi A., Morgante M., Frullone S., Marro A., Rizzo M., Martinelli G., Boccalone E., De Luca A., Santulli G. (2022). Hyperglycemia and Physical Impairment in Frail Hypertensive Older Adults. Front. Endocrinol..

[39] Mone P., Gambardella J., Lombardi A., Pansini A., De Gennaro S., Leo A.L., Famiglietti M., Marro A., Morgante M., Frullone S. (2022). Correlation of physical and cognitive impairment in diabetic and hypertensive frail older adults. Cardiovasc. Diabetol..

[40] Fung E., Lui L.T., Huang L., Cheng K.F., Lau G.H.W., Chung Y.T., Ahmadabadi B.N., Xie S., Lee J.S.W., Hui E. (2021). Characterising frailty, metrics of continuous glucose monitoring, and mortality hazards in older adults with type 2 diabetes on insulin therapy (HARE): A prospective, observational cohort study. Lancet Healthy Longev..

[41] Shah S.H., Sun J.L., Stevens R.D., Bain J.R., Muehlbauer M.J., Pieper K.S., Haynes C., Hauser E.R., Kraus W.E., Granger C.B. (2012). Baseline metabolomic profiles predict cardiovascular events in patients at risk for coronary artery disease. Am. Heart J..

[42] Moissl A.P., Lorkowski S., Meinitzer A., Pilz S., Scharnagl H., Delgado G.E., Kleber M.E., Krämer B.K., Pieske B., Grübler M.R. (2023). Association of branched-chain amino acids with mortality-the Ludwigshafen Risk and Cardiovascular Health (LURIC) study. iScience.

[43] Reisinger A.C., Posch F., Hackl G., Marsche G., Sourij H., Bourgeois B., Eller K., Madl T., Eller P. (2021). Branched-Chain Amino Acids Can Predict Mortality in ICU Sepsis Patients. Nutrients.

[44] Luo S., Surapaneni A., Rebholz C.M., Appel L.J., Coresh J., Grams M.E. (2023). Circulating Banched-Chain Amino Acids, Incident Cardiovascular Disease, and Mortality in the African American Study of Kidney Disease and Hypertension. Circ Genom Precis Med.

[45] Vanweert F., Neinast M., Tapia E.E., van de Weijer T., Hoeks J., Schrauwen-Hinderling V.B., Blair M.C., Bornstein M.R., Hesselink M.K.C., Schrauwen P. (2022). A randomized placebo-controlled clinical trial for pharmacological activation of BCAA catabolism in patients with type 2 diabetes. Nat. Commun..

[46] Liu S., Li Y., Zeng X., Wang H., Yin P., Wang L., Liu Y., Liu J., Qi J., Ran S. (2019). Burden of cardiovascular diseases in China, 1990–2016: Findings from the 2016 Global Burden of Disease Study. JAMA Cardiol.

[47] Li J.J., Liu H.H., Li S. (2022). Landscape of cardiometabolic risk factors in Chinese population: A narrative review. Cardiovasc. Diabetol..

[48] Roth G.A., Mensah G.A., Johnson C.O., Addolorato G., Ammirati E., Baddour L.M., Barengo N.C., Beaton A.Z., Benjamin E.J., Benziger C.P. (2020). Global burden of cardiovascular diseases and risk factors, 1990–2019: Update from the GBD 2019 study. J. Am. Coll. Cardiol..

[49] Tsao C.W., Aday A.W., Almarzooq Z.I., Alonso A., Beaton A.Z., Bittencourt M.S., Boehme A.K., Buxton A.E., Carson A.P., Commodore-Mensah Y. (2022). Heart disease and stroke statistics—2022 update: A report from the American Heart Association. Circulation.

[50] Yap I.K., Brown I.J., Chan Q., Wijeyesekera A., Garcia-Perez I., Bictash M., Loo R.L., Chadeau-Hyam M., Ebbels T., De Iorio M. (2010). Metabolome-wide association study identifies multiple biomarkers that discriminate north and south Chinese populations at differing risks of cardiovascular disease: INTERMAP study. J. Proteome Res..

[51] Wang F.H., Liu J., Deng Q.J., Qi Y., Wang M., Wang Y., Zhang X.G., Zhao D. (2019). Association between plasma essential amino acids and atherogenic lipid profile in a Chinese population: A cross-sectional study. Atherosclerosis.

[52] Fukushima K., Harada S., Takeuchi A., Kurihara A., Iida M., Fukai K., Kuwabara K., Kato S., Matsumoto M., Hirata A. (2019). Association between dyslipidemia and plasma levels of branched-chain amino acids in the Japanese population without diabetes mellitus. J. Clin. Lipidol..

[53] Gojda J., Cahova M. (2021). Gut Microbiota as the Link between Elevated BCAA Serum Levels and Insulin Resistance. Biomolecules.

