# Supplementary material for: Divergent Survival Outcomes Associated with Elevated Branched-Chain Amino Acid Levels among Older Adults with or without Hypertension and Diabetes: A Validated, Prospective, Longitudinal Follow-Up Study
Source: Biomolecules. 2023 Aug 16;13(8):1252. doi: 10.3390/biom13081252 (PMC10452866; doi:10.3390/biom13081252)

## Supplementary Materials

**Divergent survival outcomes associated with elevated branched-chain amino acid levels among older adults with or without hypertension and diabetes: a validated, prospective, longitudinal follow-up study**


Table S1: Cardiovascular deaths classified by ICD-10 codes among 2997 older adults during a median follow-up of 17 years .......... 2

Table S2: Non-cardiovascular deaths classified by ICD-10 codes among 2997 older adults during a median follow-up of 17 years .......... 3

Table S3: Serum branched-chain amino acid levels were consistently higher in older men than women at both time points, 0Y and 14Y.......... 5

Table S4: Adjusted Cox regression modelling of baseline independent variables on incident all-cause mortality stratified by the combined status of hypertension (HT) and diabetes (DM) with adjustment for age, sex, BMI, smoking, frailty, eGFR, and use of antihypertensive and/or hypoglycaemic drugs. .......... 6

Table S5: Details pertinent to the selection of best fitting models from restricted cubic spline and adjusted Cox regression of BCAA on all-cause, non-cardiovascular and cardiovascular mortality in older adults stratified by hypertension (HT) or diabetes (DM) status .......... 7

Table S6: Details pertinent to the selection of best fitting models from restricted cubic spline and adjusted Cox regression of BCAA on all-cause, non-cardiovascular and cardiovascular mortality in older adults stratified by the combined status of hypertension (HT) and diabetes mellitus (DM).......... 8

Figure S1: Restricted cubic splines of log(BCAA) modelled on all-cause, non-cardiovascular and cardiovascular mortality in older adults stratified by the combined status of hypertension (HT) and diabetes mellitus (DM). ..........9

**Table S1**. Cardiovascular deaths classified by ICD-10 codes among 2997 older adults during a median follow-up of 17 years.

| | Count (n) | % Cardiovascular deaths |
|---|---|---|
| Ischaemic heart disease (IHD) | 109 | 41.4% |
| Stroke | 77 | 29.3% |
| Heart failure (HF) | 29 | 11.0% |
| IHD + stroke + HF | 215 | 81.7% |
| Total cardiovascular deaths | 263 | 100% |

| ICD-10 code | Description | Count (n) |
|---|---|---|
| D65 | Disseminated intravascular coagulation [defibrination syndrome] | 1 |
| I09.9 | Rheumatic heart disease, unspecified | 2 |
| I10 | Essential (primary) hypertension | 4 |
| I11.0 | Hypertensive heart disease with (congestive) heart failure | 1 |
| I11.9 | Hypertensive heart disease without (congestive) heart failure | 17 |
| I12.0 | Hypertensive renal disease with renal failure | 1 |
| I21.9 | Acute myocardial infarction, unspecified | 40 |
| I24.9 | Acute ischaemic heart disease, unspecified | 15 |
| I25.1 | Atherosclerotic heart disease | 4 |
| I25.9 | Chronic ischaemic heart disease, unspecified | 50 |
| I26.9 | Pulmonary embolism without mention of acute cor pulmonale | 1 |
| I34.0 | Mitral (valve) insufficiency | 1 |
| I35.0 | Aortic (valve) stenosis | 2 |
| I38 | Endocarditis, valve unspecified | 3 |
| I42.0 | Dilated cardiomyopathy | 2 |
| I48.0 | Atrial fibrillation and flutter | 1 |
| I50.0 | Congestive heart failure | 23 |
| I50.9 | Heart failure, unspecified | 3 |
| I60.9 | Subarachnoid haemorrhage, unspecified | 8 |
| I61.3 | Intracerebral haemorrhage in brain stem | 1 |
| I61.5 | Intracerebral haemorrhage, intraventricular | 1 |
| I61.9 | Intracerebral haemorrhage, unspecified | 14 |
| I62.0 | Nontraumatic subdural haemorrhage | 3 |
| I62.9 | Intracranial haemorrhage (nontraumatic), unspecified | 13 |
| I63.2 | Cerebral infarction due to unspecified occlusion or stenosis of precerebral arteries | 1 |
| I63.9 | Cerebral infarction, unspecified | 2 |
| I64 | Stroke, not specified as haemorrhage or infarction | 22 |
| I67.9 | Cerebrovascular disease, unspecified | 6 |
| I69.4 | Sequelae of stroke, not specified as haemorrhage or infarction | 5 |
| I69.8 | Sequelae of other and unspecified cerebrovascular diseases | 1 |
| I71.0 | Dissection of aorta [any part] | 3 |
| I71.1 | Thoracic aortic aneurysm, ruptured | 1 |
| I71.3 | Abdominal aortic aneurysm, ruptured | 4 |
| I71.4 | Abdominal aortic aneurysm, without mention of rupture | 1 |
| I72.8 | Aneurysm and dissection of other specified arteries | 1 |
| I99 | Other and unspecified disorders of circulatory system | 1 |
| K55.9 | Vascular disorder of intestine, unspecified | 4 |
| | **Total number of cardiovascular deaths** | **263** |

**Table S2**. Non-cardiovascular deaths classified by ICD-10 codes among 2997 older adults during a median follow-up of 17 years.

| | Count (n) | % Non-cardiovascular deaths |
|---|---|---|
| Cancer/neoplasia | 375 | 38.6% |
| Infection | 383 | 39.4% |
| Miscellaneous | 214 | 22.0% |
| Total non-cardiovascular deaths | 972 | 100% |

| ICD-10 code | Description | Count (n) |
|---|---|---|
| A09.0 | Other and unspecified gastroenteritis and colitis of infectious origin | 1 |
| A09.9 | Gastroenteritis and colitis of unspecified origin | 2 |
| A16.1 | Tuberculosis of lung, bacteriological and histological examination not done | 1 |
| A16.2 | Tuberculosis of lung, without mention of bacteriological or histological confirmation | 1 |
| A16.9 | Respiratory tuberculosis unspecified, without mention of bacteriological or histological confirmation | 1 |
| A41.5 | Sepsis due to other Gram-negative organisms | 1 |
| A41.9 | Sepsis, unspecified | 25 |
| B34.8 | Other viral infections of unspecified site | 1 |
| C02.9 | Malignant neoplasm of tongue, unspecified | 1 |
| C11.9 | Malignant neoplasm of nasopharynx, unspecified | 3 |
| C15.9 | Malignant neoplasm of oesophagus, unspecified | 8 |
| C16.9 | Malignant neoplasm of stomach, unspecified | 16 |
| C17.0 | Malignant neoplasm of duodenum | 2 |
| C18.0 | Malignant neoplasm of caecum | 1 |
| C18.2 | Malignant neoplasm of ascending colon | 1 |
| C18.5 | Malignant neoplasm of splenic flexure | 1 |
| C18.7 | Malignant neoplasm of sigmoid colon | 8 |
| C18.9 | Malignant neoplasm of colon, unspecified | 53 |
| C19 | Malignant neoplasm of rectosigmoid junction | 1 |
| C20 | Malignant neoplasm of rectum | 12 |
| C22.0 | Malignant neoplasm of liver | 12 |
| C22.1 | Malignant neoplasm of intrahepatic bile ducts | 11 |
| C22.9 | Malignant neoplasm of liver and intrahepatic bile ducts, unspecified | 5 |
| C23 | Malignant neoplasm of gallbladder | 4 |
| C24.1 | Malignant neoplasm of ampulla of Vater | 1 |
| C24.9 | Malignant neoplasm of other and unspecified parts of biliary tract, unspecified | 1 |
| C25.0 | Malignant neoplasm of head of pancreas | 1 |
| C25.4 | Malignant neoplasm of endocrine pancreas | 1 |
| C25.9 | Malignant neoplasm of pancreas, unspecified | 13 |
| C26.9 | Malignant neoplasm of ill-defined sites within the digestive system | 1 |
| C31.0 | Malignant neoplasm of maxillary sinus | 1 |
| C34.9 | Malignant neoplasm of bronchus and lung, unspecified | 101 |
| C49.9 | Malignant neoplasm of other connective and soft tissue, unspecified | 2 |
| C50.9 | Malignant neoplasm of breast, unspecified | 5 |
| C53.9 | Malignant neoplasms of cervix uteri, unspecified | 1 |
| C55 | Malignant neoplasm of uterus, part unspecified | 1 |
| C56 | Malignant neoplasm of ovary | 2 |
| C61 | Malignant neoplasm of prostate | 24 |
| C64 | Malignant neoplasm of kidney, except renal pelvis | 5 |
| C67.9 | Malignant neoplasm of bladder, unspecified | 8 |
| C73 | Malignant neoplasm of thyroid gland | 2 |
| C78.0 | Secondary malignant neoplasm of lung | 1 |
| C78.7 | Secondary malignant neoplasm of liver and intrahepatic bile duct | 1 |
| C79.3 | Secondary malignant neoplasm of brain and cerebral meninges | 1 |
| C80 | Malignant neoplasm, primary site unknown, so stated | 3 |
| C80.9 | Malignant neoplasm, primary site unspecified | 13 |
| C82.9 | Follicular lymphoma, unspecified | 1 |
| C85.9 | Other and unspecified types of non-Hodgkin lymphoma, unspecified | 14 |
| C90.0 | Multiple myeloma | 3 |
| C90.1 | Plasma cell leukaemia | 1 |
| C92.0 | Acute myeloblastic leukaemia | 6 |
| C92.7 | Other myeloid leukaemia | 1 |
| C95.0 | Acute leukaemia of unspecified cell type | 1 |
| C95.9 | Leukaemia, unspecified | 2 |
| C97 | Malignant neoplasms of independent (primary) multiple sites | 3 |
| D37.6 | Neoplasm of uncertain or unknown behaviour of liver, gallbladder and bile ducts | 2 |
| D38.1 | Neoplasm of uncertain or unknown behaviour of trachea, bronchus and lung | 1 |
| D43.2 | Neoplasm of uncertain or unknown behaviour of brain, unspecified | 6 |
| D46.9 | Myelodysplastic syndrome, unspecified | 3 |
| D47.1 | Chronic myeloproliferative disease | 1 |
| D48.3 | Neoplasm of Retroperitoneum | 1 |
| E14.2 | Unspecified diabetes mellitus with renal complications | 2 |
| E14.9 | Unspecified diabetes mellitus without complications | 2 |
| E87.7 | Fluid overload | 1 |

| | | |
|---|---|---|
| F01.9 | Vascular dementia, unspecified | 2 |
| F03 | Unspecified dementia | 25 |
| G09 | Sequelae of inflammatory diseases of central nervous system | 1 |
| G12.2 | Motor neuron disease | 1 |
| G20 | Parkinson disease | 2 |
| G30.9 | Alzheimer disease, unspecified | 1 |
| G37.3 | Acute transverse myelitis in demyelinating disease of central nervous system | 1 |
| G93.4 | Encephalopathy, unspecified | 1 |
| G95.9 | Disease of spinal cord, unspecified | 1 |
| J10.0 | Influenza with pneumonia, seasonal influenza virus identified | 2 |
| J18.0 | Bronchopneumonia, unspecified | 1 |
| J18.9 | Pneumonia, unspecified | 260 |
| J44.0 | Chronic obstructive pulmonary disease with acute lower respiratory infection | 9 |
| J44.8 | Other specified chronic obstructive pulmonary disease | 1 |
| J44.9 | Chronic obstructive pulmonary disease, unspecified | 15 |
| J45.9 | Asthma, unspecified | 3 |
| J47 | Bronchiectasis | 9 |
| J64 | Unspecified pneumoconiosis | 2 |
| J69.0 | Pneumonitis due to food and vomit | 10 |
| J80 | Adult respiratory distress syndrome | 1 |
| J84.1 | Other interstitial pulmonary diseases with fibrosis | 8 |
| J84.9 | Interstitial pulmonary disease, unspecified | 1 |
| J98.4 | Other disorders of lung | 1 |
| K25.4 | Gastric ulcer | 1 |
| K26.3 | Duodenal ulcer | 1 |
| K56.6 | Other and unspecified intestinal obstruction | 4 |
| K63.1 | Perforation of intestine (nontraumatic) | 2 |
| K65.0 | Acute peritonitis | 1 |
| K65.9 | Peritonitis, unspecified | 2 |
| K72.9 | Hepatic failure, unspecified | 6 |
| K74.3 | Primary biliary cirrhosis | 1 |
| K74.6 | Other and unspecified cirrhosis of liver | 5 |
| K75.0 | Abscess of liver | 1 |
| K81.0 | Acute cholecystitis | 1 |
| K81.9 | Cholecystitis, unspecified | 1 |
| K83.0 | Cholangitis | 11 |
| K85.9 | Acute pancreatitis, unspecified | 3 |
| K92.2 | Gastrointestinal haemorrhage, unspecified | 3 |
| L12.0 | Bullous pemphigoid | 1 |
| L89.9 | Decubitus ulcer and pressure area, unspecified | 3 |
| M00.9 | Pyogenic arthritis, unspecified | 2 |
| M35.1 | Other systemic involvement of connective tissue | 1 |
| M41.9 | Scoliosis, unspecified | 1 |
| M46.9 | Inflammatory spondylopathy, unspecified | 1 |
| M81.9 | Osteoporosis, unspecified | 2 |
| N03.9 | Chronic nephritic syndrome | 3 |
| N17.9 | Acute renal failure, unspecified | 7 |
| N18.0 | Chronic kidney disease | 2 |
| N18.5 | Chronic kidney disease, stage 5 | 27 |
| N18.9 | Chronic kidney disease, unspecified | 10 |
| N19 | Unspecified kidney failure | 7 |
| N39.0 | Urinary tract infection, site not specified | 21 |
| N40 | Hyperplasia of prostate | 1 |
| N49.2 | Inflammatory disorders of scrotum | 1 |
| R02 | Gangrene, not elsewhere classified | 1 |
| R09.8 | Other specified symptoms and signs involving the circulatory and respiratory systems | 1 |
| R54 | Senility | 31 |
| R99 | Other ill-defined and unspecified causes of mortality | 4 |
| S06.2 | Diffuse brain injury | 2 |
| S06.5 | Traumatic subdural haemorrhage | 6 |
| S09.9 | Unspecified injury of head | 5 |
| S14.1 | Other and unspecified injuries of cervical spinal cord | 1 |
| S32.5 | Fracture of pubis | 1 |
| S42.4 | Fracture of lower end of humerus | 1 |
| S72.0 | Fracture of neck of femur | 1 |
| T07 | Unspecified multiple injuries | 4 |
| T17.9 | Foreign body in respiratory tract, part unspecified | 3 |
| T71 | Asphyxiation | 1 |
| T75.1 | Drowning and nonfatal submersion | 2 |
| | ICD-10 code unavailable | 5 |
| | **Total number of non-cardiovascular deaths** | **972** |

**Table S3**. Serum branched-chain amino acid levels were consistently higher in older men than women at both time points, 0Y and 14Y.

**Baseline (0Y)**

| | Men | Women | p value |
|---|---|---|---|
| N (%) | 1424 (47.5%) | 1573 (52.5%) | - |
| Total BCAA, µmol/L | 541.6 (491.3–600.4) | 489.8 (440.6–543.7) | <0.0001 |
| Isoleucine, µmol/L | 83.4 (74.7–94.7) | 74.7 (66.4–83.5) | <0.0001 |
| Leucine, µmol/L | 155.7 (140.3–173.3) | 139.3 (124.9–153.7) | <0.0001 |
| Valine, µmol/L | 302.0 (272.9–335.6) | 276.1 (248.2–308.5) | <0.0001 |

**14Y**

| | Men | Women | p value |
|---|---|---|---|
| N (%) | 408 (47.4%) | 452 (52.6%) | - |
| Total BCAA, µmol/L | 438.0 (397.2–478.0) | 407.3 (363.6–459.7) | <0.0001 |
| Isoleucine, µmol/L | 59.3 (50.6–66.9) | 49.7 (42.6–57.6) | <0.0001 |
| Leucine, µmol/L | 132.1 (120.2–145.8) | 120.9 (107.1–136.4) | <0.0001 |
| Valine, µmol/L | 245.5 (224.9–268.8) | 234.8 (212.7–266.4) | 0.0005 |

BCAA, branched-chain amino acid. Median BCAA concentrations (IQR) are shown.

**Table S4**. Adjusted Cox regression modelling of baseline independent variables on incident all-cause mortality stratified by the combined status of hypertension (HT) and diabetes (DM) with adjustment for age, sex, BMI, smoking, frailty, eGFR, and use of antihypertensive and/or hypoglycaemic drugs.

| | **$HT^+DM^+$ (deaths, n=172/289)** | | **$HT^-DM^+$ (deaths, n=62/136)** | | **$HT^+DM^-$ (deaths, n=424/993)** | | **$HT^-DM^-$ (deaths, n=577/1579)** | |
|---|---|---|---|---|---|---|---|---|
| Variable | HR (95% CI) | p value | HR (95% CI) | p value | HR (95% CI) | p value | HR (95% CI) | p value |
| Age | 1.07 (1.03-1.1) | 0.0006 | 1.19 (1.12-1.26) | <0.0001 | 1.1 (1.08-1.12) | <0.0001 | 1.1 (1.08-1.12) | <0.0001 |
| Sex | 1.59 (1.08-2.35) | 0.020 | 1.29 (0.62-2.67) | 0.50 | 1.45 (1.12-1.87) | 0.0047 | 1.77 (1.42-2.2) | <0.0001 |
| BMI | 0.92 (0.87-0.97) | 0.0033 | 0.96 (0.87-1.07) | 0.50 | 1.03 (1-1.07) | 0.062 | 0.98 (0.95-1.01) | 0.14 |
| Previous smoking | 0.99 (0.67-1.46) | 0.95 | 1.96 (1.05-3.64) | 0.034 | 1.46 (1.15-1.87) | 0.0022 | 1.23 (1-1.51) | 0.052 |
| Active smoking | 1.24 (0.57-2.68) | 0.59 | 6.23 (2-19.43) | 0.0016 | 3.09 (2-4.77) | <0.0001 | 1.72 (1.29-2.3) | 0.0003 |
| Frailty index | 3.62 (0.34-38.44) | 0.29 | 0.98 (0.01-147.47) | 0.99 | 3.22 (0.67-15.4) | 0.14 | 4.18 (0.93-18.76) | 0.062 |
| PASE | 1 (0.99-1) | 0.15 | 1 (0.99-1) | 0.48 | 1 (1-1) | 0.66 | 1 (1-1) | 0.22 |
| eGFR | 0.98 (0.97-0.99) | 0.0011 | 1.01 (0.98-1.03) | 0.62 | 0.99 (0.98-1) | 0.0020 | 0.99 (0.98-1) | 0.0066 |
| log(BCAA) ‡ | 1.52 (0.5-4.61) | 0.46 | 0.3 (0.03-3.01) | 0.30 | 0.39 (0.19-0.81) | 0.012 | 0.41 (0.23-0.73) | 0.0026 |
| Antihypertensive drugs* | 0.88 (0.58-1.35) | 0.57 | 1.94 (1.02-3.71) | 0.043 | 1.24 (0.97-1.59) | 0.082 | 1.21 (0.93-1.58) | 0.15 |
| Hypoglycaemic drugs† | 1.29 (0.87-1.91) | 0.21 | 1.14 (0.63-2.08) | 0.66 | 1.78 (0.73-4.38) | 0.21 | 1.93 (0.27-13.95) | 0.52 |

BCAA, branched-chain amino acid. BMI, body mass index. DM, diabetes mellitus. eGFR, estimated glomerular filtration rate by CKD-EPI formula. HR, hazard ratio. HT, hypertension. PASE, Physical Activity Scale for the Elderly

*Antihypertensive drugs include α-blocker, angiotensin-converting enzyme inhibitor/angiotensin receptor blocker, β-blocker, calcium channel blocker, diuretics and nitrate.

†Hypoglycaemic drugs include metformin, glibenclamide, gliclazide, glipizide, acarbose, insulin, and tolbutamide. See also Table 1.

‡HR represents 1 unit increase in log scale which is approximately equivalent to 2.718 times increase in BCAA level. See Methods for details.

Deaths (n) among total number of study participants in subgroup within parentheses.

**Table S5**. Details pertinent to the selection of best fitting models from restricted cubic spline and adjusted Cox regression of BCAA on all-cause, non-cardiovascular and cardiovascular mortality in older adults stratified by hypertension (HT) or diabetes mellitus (DM) status.

**All-cause mortality**

| | Linear model | | RCS model | | |
|---|---|---|---|---|---|
| | p value | AIC | Overall p value | Non-linear p value | AIC |
| All | <0.0001 | 18392.62 | <0.0001 | 0.076 | 18391.55* |
| $HT^+$ | 0.057 | 7880.64 | 0.010 | 0.057 | 7877.65* |
| $HT^-$ | 0.0002 | 8810.63* | 0.0009 | 0.90 | 8812.62 |
| $DM^+$ | 0.31 | 2577.85 | 0.032 | 0.017 | 2574.53* |
| $DM^-$ | <0.0001 | 14629.18* | 0.0001 | 0.47 | 14630.67 |

**Non-cardiovascular mortality**

| | Linear model | | RCS model | | |
|---|---|---|---|---|---|
| | p value | AIC | Overall p value | Non-linear p value | AIC |
| All | 0.0001 | 14453.21 | <0.0001 | 0.027 | 14450.48* |
| $HT^+$ | 0.054 | 6102.83 | 0.018 | 0.045 | 6100.97* |
| $HT^-$ | 0.0008 | 7010.43* | 0.0012 | 0.25 | 7011.15 |
| $DM^+$ | 0.33 | 1938.11 | 0.047 | 0.025 | 1935.42* |
| $DM^-$ | 0.0001 | 11607.23* | 0.0001 | 0.15 | 11607.25 |

**Cardiovascular mortality**

| | Linear model | | RCS model | | |
|---|---|---|---|---|---|
| | p value | AIC | Overall p value | Non-linear p value | AIC |
| All | 0.11 | 3943.2* | 0.27 | 0.67 | 3945.02 |
| $HT^+$ | 0.66 | 1786.94* | 0.52 | 0.29 | 1787.88 |
| $HT^-$ | 0.11 | 1803.6 | 0.051 | 0.050 | 1801.27* |
| $DM^+$ | 0.70 | 652.13* | 0.66 | 0.42 | 653.51 |
| $DM^-$ | 0.84 | 3024.78* | 0.12 | 0.22 | 3025.2 |

AIC, Akaike information criterion. RCS, restricted cubic spline. *The best-fitting model was selected based on the lowest AIC value.
p values and AIC were calculated using both linear and restricted cubic spline models (see also Figure 3).

**Table S6**. Details pertinent to the selection of best fitting models from restricted cubic spline and adjusted Cox regression of BCAA on all-cause, non-cardiovascular and cardiovascular mortality in older adults stratified by the combined status of hypertension (HT) and diabetes mellitus (DM).

**All-cause mortality**

| | Linear model | | RCS model | | |
|---|---|---|---|---|---|
| | p value | AIC | Overall p value | Non-linear p value | AIC |
| All | <0.0001 | 18392.62 | <0.0001 | 0.076 | 18391.55* |
| $HT^{+}DM^{+}$ | 0.82 | 1773.34 | 0.0613 | 0.019 | 1770.19* |
| $HT^{-}DM^{+}$ | 0.16 | 528.83* | 0.3295 | 0.68 | 530.66 |
| $HT^{+}DM^{-}$ | 0.041 | 5399.2* | 0.8594 | 0.45 | 5400.63 |
| $HT^{-}DM^{-}$ | 0.0002 | 7877.18* | 0.001 | 0.84 | 7879.14 |

**Non-cardiovascular mortality**

| | Linear model | | RCS model | | |
|---|---|---|---|---|---|
| | p value | AIC | Overall p value | Non-linear p value | AIC |
| All | 0.0001 | 14453.21 | <0.0001 | 0.027 | 14450.48* |
| $HT^{+}DM^{+}$ | 0.44 | 1323.09 | 0.048 | 0.020 | 1320.13* |
| $HT^{-}DM^{+}$ | 0.74 | 402.57* | 0.94 | 0.93 | 404.56 |
| $HT^{+}DM^{-}$ | 0.098 | 4236.68* | 0.17 | 0.41 | 4238.01 |
| $HT^{-}DM^{-}$ | 0.0003 | 6296.31* | 0.0006 | 0.34 | 6297.41 |

**Cardiovascular mortality**

| | Linear model | | RCS model | | |
|---|---|---|---|---|---|
| | p value | AIC | Overall p value | Non-linear p value | AIC |
| All | 0.11 | 3943.2* | 0.27 | 0.67 | 3945.02 |
| $HT^{+}DM^{+}$ | 0.41 | 458.17* | 0.65 | 0.68 | 460.01 |
| $HT^{-}DM^{+}$ | 0.03 | 134.62* | 0.093 | 0.87 | 136.6 |
| $HT^{+}DM^{-}$ | 0.21 | 1171.58* | 0.45 | 0.97 | 1173.58 |
| $HT^{-}DM^{-}$ | 0.33 | 1584.01 | 0.21 | 0.13 | 1583.55* |

AIC, Akaike information criterion. RCS, restricted cubic spline. *The best-fitting model was selected based on the lowest AIC value. p values and AIC were calculated using both linear and restricted cubic spline models (see also Figure S1).

**Figure S1**. Restricted cubic splines of log(BCAA) from adjusted Cox regression were modelled on A) all-cause, B) non-cardiovascular and C) cardiovascular mortality in older adults stratified by the combined status of hypertension (HT) and diabetes mellitus (DM). The best fitting model with the lowest AIC value was selected. Models were adjusted for age, sex, smoking, frailty index, eGFR, and use of antihypertensive and hypoglycaemic drugs. HR, hazard ratio. '+' and '–' denote presence and absence, respectively, of disease.

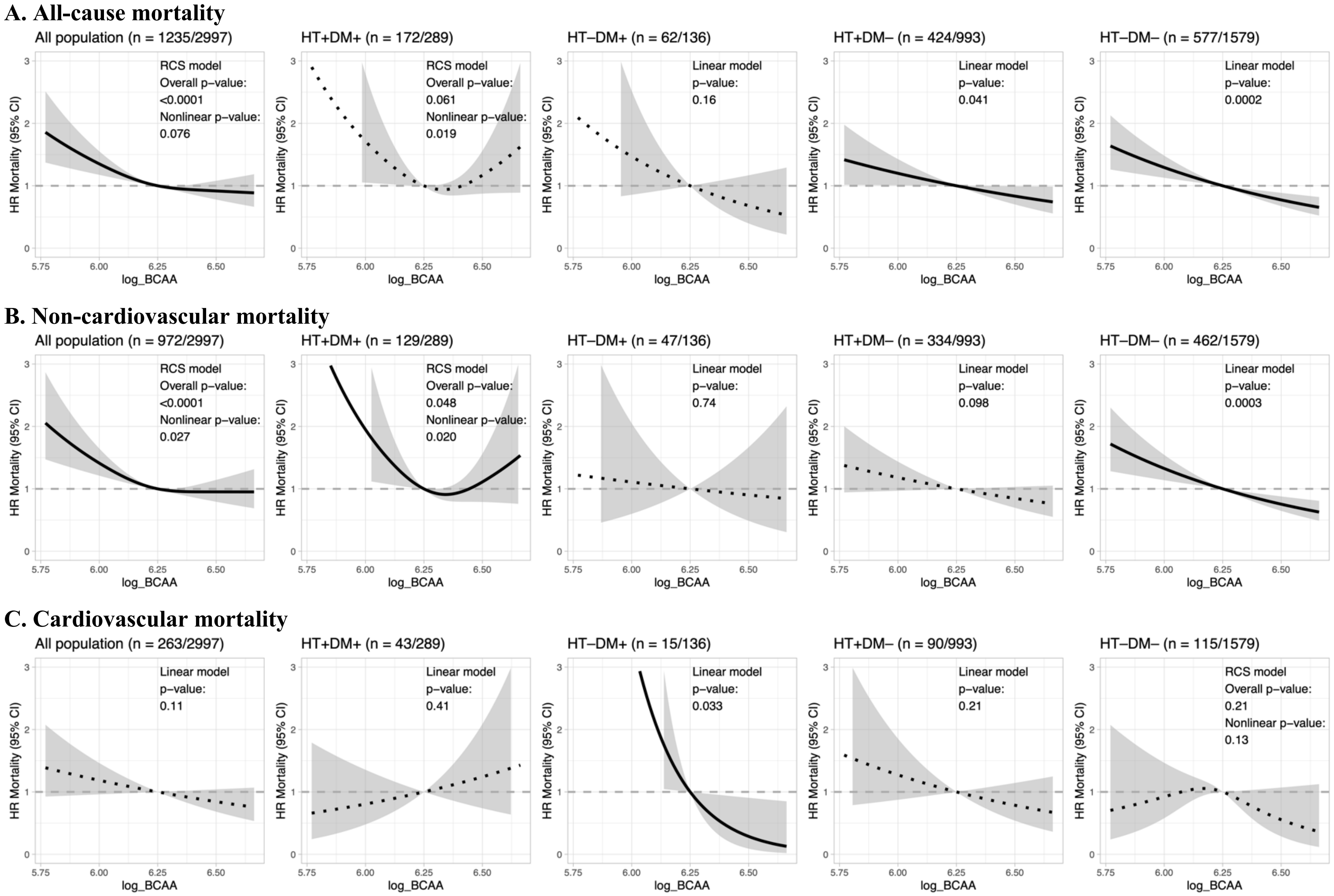

Supplement: Supplementary file 1 [file biomolecules-13-01252-s001.zip › biomolecules-2478160-supplementary.pdf]
